# Synthesis, Biological Evaluation, Molecular Dynamics, and QM-MM Calculation of Spiro-Acridine Derivatives Against Leishmaniasis

**DOI:** 10.3390/microorganisms13061297

**Published:** 2025-06-02

**Authors:** Sonaly Albino, Michelangela Nobre, Jamire da Silva, Malu dos Reis, Maria Nascimento, Mayara de Oliveira, Tatiana Borges, Lucas Albuquerque, Selma Kuckelhaus, Luis Alves, Fábio dos Santos, Maria de Lima, Igor Nascimento, Teresinha da Silva, Ricardo de Moura

**Affiliations:** 1 Postgraduate Program in Therapeutic Innovation, Federal University of Pernambuco, Recife 50740-570, Brazil; sonaly.albino@hotmail.com (S.A.); teresinha100@gmail.com (T.d.S.); 2 Drug Development and Synthesis Laboratory, Department of Pharmacy, State University of Paraíba, Campina Grande 58429-500, Brazil; michelysuelleny@gmail.com (M.N.); jamiremuriel@hotmail.com (J.d.S.); malureis_farmacia@hotmail.com (M.d.R.); maria.veronica@aluno.uepb.edu.br (M.N.); 3 Morphology Area, Faculty of Medicine-UnB, University of Brasília, Darcy Ribeiro Campus, Brasília 70910-900, Brazil; mayaragco@gmail.com (M.d.O.); selmask@gmail.com (S.K.); 4 Cellular Immunology Laboratory, Pathology Area, Faculty of Medicine, University of Brasília, Darcy Ribeiro Campus, Brasília 70910-900, Brazil; tatianakarlab@gmail.com (T.B.); lucasffriaca@gmail.com (L.A.); 5 Department of Parasitology, Aggeu Magalhães Institute (FIOCRUZ/PE), Recife 50740-465, Brazil; lcalves@cpqam.fiocruz.br (L.A.); brayner@cpqam.fiocruz.br (F.d.S.); 6 Department of Antibiotics, Federal University of Pernambuco, Biosciences Center, Recife 50740-570, Brazil; nenalima.mariadocarmo@gmail.com; 7 Postgraduate Program of Pharmaceutical Sciences, Pharmacy Department, State University of Paraíba, Campina Grande 58429-500, Brazil

**Keywords:** spontaneous cyclization, molecular simplification, leishmaniasis, immunomodulation, structure–activity relationship, QM-MM

## Abstract

Leishmaniasis is a neglected tropical disease caused by *Leishmania* sp. The therapeutic arsenal is reduced and limited. In this context, acridine derivatives present themselves as promising leishmanicidal compounds. This paper involved synthesizing and evaluating the antileishmanial and immunomodulatory potential of spiro-acridine derivatives. Six spiro-acridine derivatives were obtained through nucleophilic substitution reactions between the acetohydrazide/acetamide intermediates and 9-carbaldehydeacridine, followed by spontaneous cyclization. IR, NMR, and HRMS confirmed the structures. These were analyzed in vitro against *L. infantum* and *L. amazonensis* to determine anti-promastigote, anti-amastigote, and cytotoxicity effects. Immunomodulatory activity was evaluated using CBA, DCF-DA, and DAF-FM diacetate. In silico evaluation included molecular docking and dynamics. The spiro-acridines showed a wide range of anti-promastigote activities (IC_50_ = 0.73–234.95 µM) and non-toxicity to red blood cells. AMTAC-02 and ACMD-03 demonstrated satisfactory anti-amastigote effect (IC_50_ = 10.47–13.50 µM), low toxicity to macrophages (CC_50_ = 27.22–569.50 µM), and cytokine and reactive species modulation. Molecular docking proposed cysteine protease B of *L. amazonensis* as a target, and molecular dynamics analysis highlighted the complex’s stability using RMSD, R_g_, SASA, DCCM, PCA, and MM-PBSA (ΔG = −65.225 kJ/mol). Furthermore, QM-MM calculation provided the best energy for ACMD-03 (−199.30 au). Hence, AMTAC-02 and ACMD-03 demonstrated antileishmanial potential, making them promising entities for the development of leishmanicidal drug candidates.

## 1. Introduction

Leishmaniases is a neglected tropical disease (NTD) caused by the genus *Leishmania* sp., an intracellular protozoa belonging to the Trypanosomatidae family [1,2]. Epidemiological statistical data published by the World Health Organization indicate that approximately 700,000 to 1 million cases occur annually, and currently, 1 billion people reside in endemic areas, putting them at imminent risk of infection [3].

*Leishmania* sp. is a heteroxenic protozoan that completes its life cycle by alternating between definitive mammalian hosts, such as humans, dogs, and rodents, and intermediate hosts, which include sandflies and mosquitoes [4]. Additionally, its development comprises converting between two morphological stages: amastigote and promastigote forms [5,6]. These protozoa, which comprise approximately 20 different species, provide several clinical forms (mucocutaneous, cutaneous, and visceral leishmaniasis), as well as immunopathological and histopathological manifestations, depending on the characteristics of the species and the host’s defense mechanism [7,8].

The host’s immunological response to the infection by the *Leishmania* sp. parasite involves a series of complex mechanisms. Various factors, including the genetic diversity of the host, the Leishmania species, and different parasitic isolates, influence it. From the inoculation of metacyclic promastigote forms into the host’s bloodstream until their effective infection, differentiation into amastigotes, and multiplication in macrophages, the action of numerous components of the innate and adaptive immune system are present in the process of eradicating the infection or enabling its progression [9,10]. Therefore, understanding the immunological factors involved and altered during the infectious process is crucial for effective parasite eradication.

Furthermore, the current treatment for several clinical forms of this disease is limited, and the therapeutic arsenal of leishmanicidal drugs has remained invariable for years. It comprises only five drugs: sodium stibogluconate, amphotericin B, miltefosine, paromycin, and pentamidine [11,12,13]. Moreover, the main limitations factors involve low efficacy, complicated administration, high cost, increasing resistance, and systemic toxicity [2,12].

Consequently, there is a need to obtain novel antileishmanial drugs that can overcome these limitations [14]. For this, methods based on computer-aided drug design, which have emerged over the years, are essential in any drug design campaign [15,16]. In addition, we highlight acridine derivatives as potential chemotherapy scaffolds that have been investigated against species of *Leishmania* sp. since the last decade of the 20th century by Werbovertz et al. [17], with the identification of the in vitro leishmanicidal potential against promastigote forms of *L. chagasi* and *L. donovani* [18,19,20,21].

More recently, Almeida et al. [22] demonstrated the in vitro activity of spiro-acridine compounds against amastigotes and promastigotes of *L. infantum*. The results demonstrated the potency of AMTAC-01 and AMTAC-11, which presented IC_50_ values of 2.039 and 1.109 μg/mL against the promastigote forms, respectively. Additionally, AMTAC−11 demonstrated excellent performance by inhibiting parasite growth 33.2% more than the control compound (amphotericin B) against amastigote forms. This spiro-acridine derivative had an EC_50_ of 0.974 μg/mL and was non-toxic to PBMC cells at concentrations above 100 μg/mL, resulting in a selectivity index of greater than 102.67.

Thus, due to the promising results obtained by Almeida et al. [22], this work proposes the design, synthesis, and investigation in silico and in vitro of the antileishmanial activity of different spiro-acridine derivatives (AMTACs and ACMDs), against both forms and two species of the parasite, to determine the leishmanicidal effect performed and the influence of these compounds on components of the immune system.

## 2. Materials and Methods

### 2.1. Chemistry

The starting materials —benzaldehyde, 4-methoxybenzaldehyde, 4-chlorobenzaldehyde, aniline, 4-methoxyaniline, and 4-chloroaniline—used for the synthesis of the intermediate reagents 2-cyano-N’-phenylacetamide and 2-cyano-N’-benzylideneacetohydrazide were obtained from Sigma Aldrich (St. Louis, MO, USA). The solvents for analysis and synthesis were supplied by Sigma-Aldrich, Fluka (Buchs, SG, Switzerland), and Merck (Darmstadt, Germany) and used without previous purification. Melting points were determined in closed capillary tubes using the device Quimis Model 340 (Quimis, Diadema, Brazil). Thin layer chromatography (TLC) was performed on Fluka Analytical silica gel 60 plates, with a thickness of 0.25 mm, and visualized under UV light (254 or 365 nm). Infrared spectra were generated with KBr disks on an IRPrestige-21 Spectrometer, Shimadzu^®^. The data were processed in the software Origin 8.0. Proton (¹H NMR) and carbon nuclear magnetic resonance (^13^C NMR) spectra were obtained on a Varian Model Plus spectrometer (Varian, Santa Clara, CA, USA) at 300 MHz, using deuterated dimethyl sulfoxide (DMSO-d_6_) as the solvent. The spectra were plotted and interpreted using MestreNova 12.0 software. Chemical shifts (δ) were expressed in parts per million (ppm), and coupling constants (*J*) were indicated in Hertz (Hz). The multiplicities of the signals were designated as simplet (s), doublet (d), or multiplet (m). Mass spectra were recorded using matrix-assisted laser desorption/ionization (MALDI) with an Autoflex III time-of-flight (TOF) mass spectrometer (Bruker Daltonics, Billerica, MA, USA).

#### 2.1.1. Procedure for Synthesis of AMTAC Derivatives

**Scheme 1:**
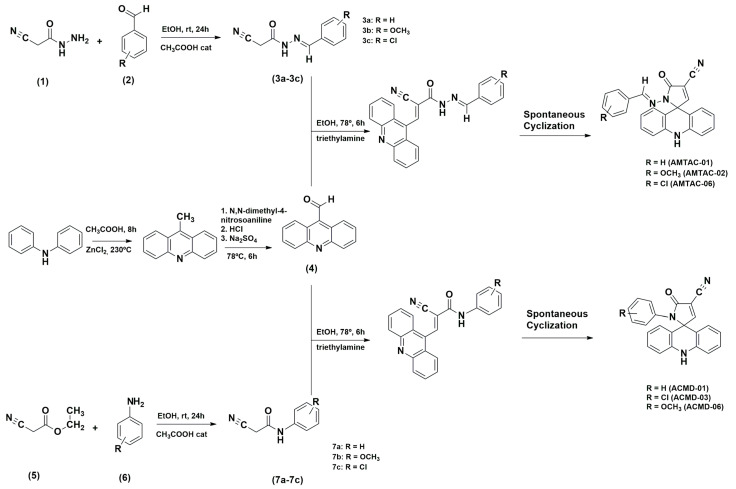
Synthetic routes were used to obtain the **AMTAC** and **ACMD** derivatives.

The 2-cyano-*N*′-benzylidene-acetohydrazide intermediates (3a–3c) were obtained through a nucleophilic addition reaction between 2-cyanoacetohydrazide (1) and several aromatic aldehydes (2) in ethanol medium and at room temperature. Then, 9-carbaldehydeacridine (4) was coupled to 2-cyano-*N*′-benzylidene-acetohydrazide intermediates by a nucleophilic substitution reaction at 78 °C in ethanolic and basic medium, followed by spontaneous cyclization according to Scheme 1. The reaction was monitored through TLC. The product was then filtered, and the obtained crystals were washed with water and then recrystallized in ethanol. These derivatives have been previously synthesized and structurally characterized by Almeida et al. [23] (AMTAC-01 and AMTAC-02) and Gouveia et al. [24] (AMTAC-06).

**AMTAC-01**: (*E*)-1′-(benzylideneamino)-5′-oxo-1′,5′-dihydro-10H-spiro[acridine-9,2′-pyrrole]-4′-carbonitrile: Green solid. Formula: C_24_H_16_N_4_O; M.W.: 376.41 g/mol; yield: 79.82%; melting point: 217–222 °C. IR (cm^−1^): 3291.85 (NH), 1705.12 (C=O), 1614.95 (C=N), 1486.49 (C=C). 1H-NMR (300 MHz, DMSO-*d6*): δ 9.85 (1H, s, NH); δ = 8.56 (1H, s, CH); δ = 8.39 (1H, s, CH); δ = 7.34 (4H, m, aromatic); δ = 7.27 (2H, m, aromatic); δ = 7.01 (4H, t, aromatic) *J* = 7.6 Hz; δ = 6.83 (2H, t, aromatic) *J* = 7.6 Hz; δ = 2.49. 13C-NMR: δ 68.79, 108.16, 121.34, 115.09, 120.42, 126.83, 127.01, 128.85, 130.63, 133.65, 138.60, 147.58, 160.64, 160.84. MS [M+H]+: calculated = 376.1324; found = 377.1240 [23].

**AMTAC-02**: (*E*)-1′-((4-methoxybenzylidene)amino)-5′-oxo-1′,5′-dihydro-10H-spiro[acridine-9,2′-pyrrole]-4′-carbonitrile: Yellow solid. Formula: C_25_H_18_N_4_O_2_; M.W.: 406.43 g/mol; yield: 63.5%; melting point: 236–240 °C. IR (cm^−1^): 1722.85 (C=O), 1609.88 (C=N), 1486.04 (C=C), 1256.97 (Ar-O-CH_3_), 1108.81 (Ar-OCH_3_). 1H-NMR (300 MHz, DMSO-*d6*): δ = 9.83 (1H, s, NH); δ = 8.51 (1H, s, CH); δ = 8.37 (1H, s, CH); δ = 3.71 (3H, s, OCH_3_); δ = 7.29 (4H, m, aromatic); δ = 7.00 (4H, m, aromatic); δ = 6.86 ppm (4H, m, aromatic); δ = 2.49. 13C-NMR: 55.21, 68.83, 108.71, 111.56, 112.41, 114.34, 115.06, 120.36, 126.20, 126.98, 128.59, 130.06, 138.61, 148.30, 160.39, 160.70, 161.12. MS [M+H]+: calculated = 406.1430; found = 407.1424 [23].

**AMTAC-06**: (*E*)1-(4-chlorobenzylideneamino)-5-oxo-1,5-dihydro-10H-spiro[acridine-9,2-pyrrole]-4-carbonitrile: Yellow powder. Formula: C_24_H_15_ClN_4_O; M.W.: 410.8551 g/mol; yield: 24%; melting point: 240–242 °C; R*f*: 0.54 (*n*-hexane/EtOAc 7:3). IR (KBr, cm^−1^): 3315.63 (NH), 3089.96 (CH), 2239.35 (CN), 1676.14 (C=O), 1485.18, 1334.74 (aromatic CN), 790.81 (Ar-Cl). 1H NMR (DMSO-*d*6): *δ* 9.82 (s, 1H, NH), 8.55 (s, 1H, CH), 8.42 (s, 1H, N=CH), 7.38 (m, 4H, aromatic), 7.26 (m, 2H, spiro-acridine), 7.00 (m, 4H, spiro-acridine), 6.82 (m, 2H, spiro-acridine). 13C NMR: *δ* 69.33, 108.58, 111.76, 112.78, 115.58, 120.90, 127.50, 128.93, 129.47, 130.65, 133.03, 135.56, 139.05, 146.84, 161.18, 161.38. HRMS *m*/*z* [M+H]+: calculated: 410.8551; found: 411.0870 [24].

#### 2.1.2. Procedure for Synthesis of ACMD Derivatives

The 2-cyano-*N*′-phenyl-acetamide intermediates (7a-7c) were obtained through an amidation reaction between ethyl 2-cyano-acetate (5) and different aromatic anilines (6) in dimethylformamide at 170 °C. Next, 9-carbaldehydeacridine (4) was coupled to 2-cyano-*N*′-phenyl-acetamide intermediates by an aromatic nucleophilic substitution reaction at 78 °C in ethanolic and basic medium, followed by spontaneous cyclization according to Scheme 1. The reaction was monitored through TLC. The product was then filtered, and the obtained crystals were washed with water and recrystallized in ethanol.

ACMD-01: (*E*)5′-oxo-1′-phenyl-1′, 5′-dihydro-10*H*-spiro{acridine-9,2′-pyrrole]-4′-carbonitrile: Yellow powder. Formula: C_23_H_15_N_3_O; M.W.: 349.1215 g/mol; yield: 75%; melting point: 240–242 °C; R*f*: 0.57 (*n*-hexane/EtOAc 6:4). IR (KBr, cm^−1^): 3314.63 (NH), 3094.96 (CH), 2235.35 (CN), 1695.14 (C=O), 1450.18, 1343.74 (aromatic CN). 1H NMR (DMSO-*d*6): *δ* 9.63 (s, 1H, NH), 8.57 (s, 1H, CH), 7.22 (m, 2H, *J* = 8.4 e *J* =1.6), 7.12 (m, 3H, aromatic), 7.07 (m, 2H, spiro-acridine), 6.85 (m, 4H, spiro-acridine), 6.76 (m, 2H, spiro-acridine). 13C NMR: *δ* 70.60, 108.90, 112.20, 113.10, 115.58, 120.69, 125.40, 127.10, 128.10, 129.01, 130.53, 136.29, 138.71, 163.30, 164.18. HRMS *m*/*z* [M+H]+: calculated: 349.3847; found: 350.1168.

ACMD-03: (*E*)1′-(4-chlorophenyl)-5′-oxo-1′, 5′-dihydro-10*H*-spiro{acridine-9,2′-pyrrole]-4′-carbonitrile: Yellow powder. Formula: C_23_H_14_ClN_3_O; M.W.: 383.0825 g/mol; yield: 31%; melting point: 252–254 °C; R*f*: 0.46 (*n*-hexane/EtOAc 7:3). IR (KBr, cm^−1^): 3312.88 (NH), 3097.57 (CH), 2242.08 (CN), 1693.76 (C=O), 1478.45, 1389.45 (aromatic CN). 1H NMR (DMSO-*d*6): *δ* 9.59 (s, 1H, NH), 8.62 (s, 1H, CH), 7,27 (m, 1H, aromatic), 7.24 (m, 2H, aromatic), 7.15 (m, 2H, aromatic), 6,92 (m, 2H, spiro-acridine), 6.87 (m, 2H, spiro-acridine), 6.84 (m, 2H, spiro-acridine). 13C NMR: *δ* 70.60, 108.83, 112.02, 113.00, 115.68, 120.79, 126.70, 128.10, 129.21, 130.58, 131.39, 135.31, 138.71, 163.30, 164.18.

ACMD-06: (*E*)1′-(4-methoxyphenyl)-5′-oxo-1′, 5′-dihydro-10*H*-spiro{acridine-9,2′-pyrrole]-4′-carbonitrile: Yellow powder. Formula: C_24_H_11_N_3_O_2_; M.W.: 379.1321 g/mol; yield: 69%; melting point: 262–265 °C; R*f*: 0.47 (*n*-hexane/EtOAc 6:4). IR (KBr, cm^−1^): 3304.07 (NH), 3092.23 (CH), 2235.35 (CN), 1679.24 (C=O), 1479.02 (aromatic CN). 1H NMR (DMSO-*d*6): *δ* 9.48 (s, 1H, NH), 8.54 (s, 1H, CH), 7,18 (m, 4H, aromatic), 6,87 (m, 4H, spiro-acridine), 6.60 (m, 4H, spiro-acridine), 3.59 (m, 3H, CH_3_). 13C NMR: *δ* 70.60, 109.89, 112.10, 113.10, 114.34, 114.61, 120.60, 127.63, 128.40, 130.41, 138.69, 163.23, 164.03. HRMS *m*/*z* [M+H]+: calculated: 379.4107; found: 379.6972

### 2.2. Experimental Biological Evaluation

#### 2.2.1. Antileishmanial Activity

##### Parasite Culture

The promastigote cultures of *L. infantum* (Strain MHOM/BR/70/BH46) and *L. amazonensis* (Strain MHOM/BR/PH8) were kept in Schneider medium (Sigma-Aldrich, St. Louis, MO, USA) supplemented with 20% fetal bovine serum (FBS) and 1% streptomycin/penicillin in a B.O.D. incubator (J. Prolab, model JP. 100 LBCM/CPAM) at 26 °C. The promastigote strains were used in the exponential growth phase in all stages for experimental purposes.

The amastigote forms of *L. amazonensis* were maintained under cryopreservation until they were transferred to an NNN medium (Novy–MacNeal–Nicolle) and incubated at 26 °C until the parasites reached the log growth phase. Subsequently, the suspension was transferred to Schneider culture medium (Sigma-Aldrich, St. Louis, MO, USA), supplemented with 20% inactivated fetal bovine serum and 0.2% gentamicin sulfate at 26 °C, causing the parasites to return to the log growth phase. The cultures were then maintained at 37 °C, producing a growth curve and the formation of the axenic amastigote strains.

##### Determination of the Inhibitory Effect in Promastigote Forms of *L. amazonensis* and *L. infantum* Strains

The Neubauer chamber method was used to assess the inhibitory concentrations of the synthesized spiro-acridines capable of eliminating 50% of the promastigotes in cultures of *L. amazonensis* and *L. infantum* (IC_50_) strains [25,26]. Promastigotes from both species were collected, counted, and diluted in Schneider’s medium (Sigma-Aldrich, St. Louis, MO, USA) supplemented with 20% fetal bovine serum (FBS) at a final concentration of 1 × 10^6^ cells/mL. The evaluated promastigotes were then exposed to different concentrations of the tested compounds (10, 5, 2.5, 1.25, and 0.625 μg/mL). The positive control used the commercial drug Amphotericin B^®^, while the negative control consisted of promastigotes incubated in Schneider’s medium alone. After 72 h of incubation at 25 °C, culture growth was observed by counting the viable cells using a Neubauer chamber and calculated using the following Equation (1):

No. Leishmania/mL = No. of cells counted × No. of Neubauer chamber(1)

quadrants × the dilution used × 10^4^ (Neubauer chamber correction factor)

The concentration parameter that inhibits 50% of parasite growth (IC_50_) was used to estimate the growth inhibition of the promastigote species of *Leishmania* sp. Thus, IC_50_ was determined by linear regression analysis with SPSS 8.0 software for Windows. Each test was performed in two independent experiments, with different cultures and in technical triplicate.

##### Determination of the Inhibitory Effect in Amastigote Anexic Forms of *L. amazonensis*

The concentrations of the compounds AMTAC-02 and ACMD-03 capable of eliminating 50% of the cells in the cultures of *L. amazonensis* amastigotes (IC_50_) were determined using the MTT [3-(4,5-dimethylthiazol-2-yl)-2,5-diphenyltetrazolium bromide] (Sigma-Aldrich) reagent. In a 96-well plate, the assay was initiated by diluting both compounds to concentrations of 2, 4, 8, 16, and 32 μg/mL and 2.0 × 10^5^ axenic amastigotes per well. The plates were incubated at 37 °C for 24 h. Then, 10 μL of MTT was added per well, and the cultures were re-incubated for 4 h. Subsequently, 50 μL of dimethyl sulfoxide was added to each well to solubilize the formazan crystals formed. The absorbances were measured using a SpectraMAX GeminiXS plate spectrophotometer (Molecular Devices LLC) at a wavelength of 570 nm. The negative control consisted of untreated cells, while the positive control was assessed by incubating the axenic amastigotes with the reference drug Amphotericin B^®^ [13].

#### 2.2.2. Determination of Cell Viability

##### Assessment of the Hemocompatibility of Spiro-Acridine Derivatives in Red Blood Cells

The hemocompatibility of the spiro-acridine derivatives was determined using human red blood peripheral cells (O+) from healthy adults. The erythrocytes were diluted in phosphate-buffered saline (PBS) to a concentration of 5% in an 80 µL suspension and subsequently incubated with 20 µL of different concentrations of spiro-acridine derivatives and Amphotericin B^®^ (400, 200, 100, 50, 25, 12,5, 6,25 and 3,12 μg/mL). Afterward, the samples were incubated for 1 h at 37 °C in a B.O.D incubator, and the reaction was stopped by adding 200 μL of PBS (1.5 mM KH_2_PO_4_, 8.1 mM Na_2_HPO_4_, 136.9 mM NaCl, and 2.6 mM KCl, pH 7.2). The samples were centrifuged at 2000 rpm for 10 min, at room temperature. The supernatants were collected and subjected to spectrophotometry using a spectrophotometer (model Biosystems ELx800, Curitiba, PR, Brazil) at 540 nm. The hemolysis percentage was determined as [(Abs_sam_ − Abs_con_)/(Abs_tot_ − Abs_con_) × 100], where Abs_sam_ refers to the absorbance of the sample, Abs_con_ to the absorbance of the blank control (without compounds), and Abs_tot_ to the absorbance of total hemolysis (obtained by replacing the sample solution with an equal volume of Milli-Q water). The results were expressed as average hemolytic concentration (HC_50_), considering the positive control as 100% hemolysis [22,27].

##### Determination of the Cytotoxic Effects in J774 Macrophage Cultures

J774A.1 macrophages (ATCC TIB-67) were kept cryopreserved in a Falcon tube containing 5 mL of DMEM (Dulbecco’s modified Eagle medium) culture medium (Gibco^®^), supplemented with 10% inactivated fetal bovine serum, 1% non-essential amino acids, and 1% gentamicin sulfate. The tube was centrifuged at 200 rpm for 10 min. Afterward, the supernatant was discarded, and the cells were resuspended in the culture medium and transferred to a culture bottle kept in an incubator at 37 °C with 5% CO_2_.

The assays were carried out in triplicate using a 96-well plate to evaluate the cytotoxic effect of the selected spiro-acridine compounds on J774 macrophage cultures. The compounds were diluted and seeded at predetermined concentrations (32, 16, 8, 4, and 2 μg/mL), parallel to the distribution of 1.0 × 10^5^ J774 macrophages per well. The plate was incubated for two hours in a 5% CO_2_ incubator at 37 °C. Only the cells were incubated in the negative control without the tested molecules. In contrast, the positive control was performed by incubating the macrophages with the commercial drug Amphotericin B^®^. Later, 10 μL of the MTT reagent was added per well, and the cultures were re-incubated for 4 h. Finally, 50 μL of DMSO was added to each well, and the absorbance was read on a SpectraMAX GeminiXS plate spectrophotometer (Molecular Devices LLC, San Jose, CA, USA) at a wavelength of 570 nm.

The 50% cytotoxic concentration in macrophages (CC_50_) was determined using non-linear regression with GraphPad Prism software (San Diego, CA, USA) version 5.01.

#### 2.2.3. Assessment of Cytokine Profile in Immunological Responses

The assessment of the influence of spiro-acridine derivatives on the cytokine profile of immunological responses was carried out by the quantification of the cytokines IL-2, IL-4, IL-6, IL-10, IL-17A, INF-γ, and TNF-α in supernatants of cell cultures of J774 macrophages infected with *L. amazonensis* amastigotes through cytometry bead assay (CBA), according to the manufacturer’s instructions (BD Bioscience, Franklin Lakes, NJ, USA). The cells were adhered to a plate, infected with amastigotes of *L. amazonensis,* and treated with the spiro-acridines under analysis in the following concentrations: 2, 4, and 8 μg/mL. After the 24-h treatment period, 50 μL of the supernatant from each well was transferred to a conical tube, while 50 μL of the capture bead mixture and 50 μL of the detection reagent were added. The samples were incubated for 2 h at room temperature and protected from light. After incubation, 500 μL of washing buffer was added to each tube, followed by centrifugation for 5 min. The supernatant was discarded, and 300 μL of washing buffer was added. A calibration curve containing the following concentrations of each cytokine was added to the experiment: 0, 20, 40, 80, 156, 312.5, 625, 1250, 2500, and 5000 pg/mL. Uninfected and untreated macrophages were used as the negative control, while the untreated macrophages infected with *L. amazonensis* were used as the positive control. The fluorescence intensity in each sample was captured on the BD LRS II FORTESSA flow cytometer using the DIVA program version 7 (BD Bioscience), and the fluorescence data was processed using the FCAP array program version 3 (BD Bioscience). The experiments were performed in triplicate [28].

#### 2.2.4. Evaluation of the Production of Reactive Oxygen and Nitrogen Species

The assay for the quantification of reactive oxygen species (ROS) and reactive nitrogen species (RNS) was executed using, respectively, the probe 2′,7′-dichlorodihydrofluorescein diacetate (DCF-DA) and 4-amino-5-methylamino-2′,7′-difluorofluorescein diacetate (DAF-FM diacetate), which produce 2–7-dichlorofluorescein (DCF) and 4-amino-5-methylamino-2′,7′-difluorofluorescein (DAF-FM) in the presence of the respective reactive species. Therefore, initially, macrophages were adhered to and infected with *L. amazonensis* in a plate, followed by the treatment with AMTAC-02 or ACMD-03 at concentrations of 2, 4, and 8 μg/mL. Subsequently, following a 24-h treatment period, the plates were incubated for 30 min with the DCF-DA probe diluted in saline (pH 7.2) and for 1 h with the DAF-FM diacetate probe diluted in saline (pH 7.2), respectively, in a humid chamber at 37 °C with 5% CO_2_, protected from light. The cells were washed twice with saline (pH 7.2) at room temperature and then resuspended in 200 µL of saline under the same conditions. The negative control comprised uninfected and untreated macrophages, while the positive control comprised untreated macrophages infected with *L. amazonensis*. Afterward, the fluorescence intensity in each sample was captured using the BD LRS II FORTESSA flow cytometer and the DIVA program version 7 (BD Bioscience). The mean fluorescence intensity (MFI) was then obtained by processing the data in FlowJoTM version 10.6.1. The experiment was performed in triplicate, and the results were calculated as the difference in MFI in each group [28].

#### 2.2.5. Statistical Analysis

The normality of the variables was assessed using the Kolmogorov–Smirnov test, while the Bartlett test was employed to evaluate the homogeneity of variances. The paired *t*-test or Wilcoxon test were employed for comparison between two normal or non-normal samples. For multiple comparisons, the ANOVA test was used. All statistical analyses and graphical representations were generated using the Prism software package. Only *p*-values below 0.05 were considered significant throughout the analyses [29].

### 2.3. Computational Experiments

#### 2.3.1. Molecular Docking Simulation

Using the Research Collaboratory for Structural Bioinformatics Protein Data Bank database (RCSB PDB) (https://www.rcsb.org/) (accessed on 29 August 2024) [30], the 3D structure of trypanothione reductase (TyR) from *Leishmania infantum* (PDB 4APN), sterol 14α-demethylase—CYP51 (PDB 3L4D), topoisomerase I of *Leishmania donovani* (PDB 2B9S), topoisomerase I from *homo sapiens* (PDB 1TL8), and cysteine protease B from *Leishmania amazonensis* (CPBLa), were built from homology modeling in a previously work [26]. All hydrogens were added for this structure, while cocrystallized ligands, ions, and water molecules were removed using the *Chimera^®^* 1.17.3 [31] software. Subsequently, these complexes were submitted to the re-docking procedure using the *GOLD^®^* 2022.3.0 software [32]. For the re-docking, all four algorithms (ChemScore, Chemical Piecewise Linear Potential (ChemPLP), Astex Statistical Potential (ASP), and GoldScore) were applied to provide FitScores and binding modes. The best binding pose was chosen for each ligand, and their root-mean-square deviation (RMSD) values were calculated in GOLD^®^ 2022.3.0 software. For the ligands, a conformational analysis was initially carried out using the *MarvinSketch* 23.16 software [33] for each compound. Thus, ten conformations were generated for each ligand, and the conformation with the lowest energy value was chosen. Finally, these structures were optimized to correct angles and bond lengths using the ArgusLab 4.0.1 software [34] by applying the semi-empirical AM1 (Austin Model 1) method. Finally, molecular docking studies were performed using the *GOLD^®^* software, employing the adequate scoring function chosen from our initial re-docking procedures. A 6 Å region around the cocrystallized ligand was selected using the maximum efficiency of the genetic algorithm (GA), and ten binding poses were generated for each ligand.

#### 2.3.2. Molecular Dynamics Simulations

After molecular docking, the CPBLa was chosen for molecular dynamics (MD) simulations using the *GROMACS^®^* 2020 software. In this manner, charges and hydrogens were added using the *Chimera* software through the *DockPrep* tool. Next, the CHARMM36 force field was used with the TIP3P solvation method. In parallel, ligand topology was generated using the web software SwissParam (http://www.swissparam.ch/, accessed on 10 November 2024) [33]. Next, a 1.0 nm triclinic box was created, with water and ions added at physiological concentrations. This was followed by the system’s equilibrium in 10,000 steps using the conjugate gradient method and its total minimization in 20,000 steps. Next, NVT (the constant number of particles, volume, and temperature) and NPT (the constant number of particles, pressure, and temperature) equilibriums were performed at a temperature of 300 K for 10 ns. The final simulation was performed at 100 ns with the free protein and complexed with ACMD-03. The RMSD, R_g_, and SASA plots were generated using the *Xmgrace^®^* 5.1.25 software. This protocol aligns with other previously published works from our research team [26,34,35,36,37,38]. In addition, the trajectory of MD simulations was used in the *Bio3D^®^* 2.3.0 software [39] to generate the principal component analysis (PCA) and dynamic cross-correlation matrix (DCCM) of the free CPBLa and in complex with the ACMD-03.

#### 2.3.3. MM-PBSA Calculations

The MM-PBSA (molecular mechanics Poisson–Boltzmann surface area) method was used to calculate the Gibbs free binding energy (Δ*G_binding_*) based on van der Waals and electrostatic (unbound) interactions between the ligand and its receptor during an MD simulation [40]. For this, the Δ*G_binding_* was calculated by the difference between the free energy of complex protein–ligand (*G_cpx_*) and unbound protein and ligand (*G_rec_*). Finally, the Δ*G_binding_* was considered, such as summation of the changes in the solvation entropy (−*T*Δ*S_sol_*), binding energy (Δ*E_bind_*), and conformational entropy (−*T*Δ*S_conf_*), as shown in Equation (2) [41], and were performed using the *g_mmpbsa* tool using the trajectory files obtained after the MD simulation at 100 ns, using the *GROMACS^®^* 2020 software. Then, Δ*G_binding_* values were determined as the average free-interaction and solvation energies [40]. This protocol is according to other works from our research group [26,34,37].

(2) $$\Delta G_{binding}=E_{binding}-T\Delta S_{sol}-T\Delta S_{conf}\;\;\;\;$$

#### 2.3.4. QM-MM Binding Free Energy Calculations

The hybrid method between quantum mechanics–molecular mechanics (QM-MM) was employed to investigate the binding free energy of the ACMD-03 and its proposed target CPBLa. For this, the GROMACS 2020 software was used with the CP2K interface. Thus, the complex was added to a cubic box of 1.0 nm, and the topology was created using the CHARMM27 force field and the TIP3P water model. The ligand topology was constructed using the web-based software SwissParam (http://www.swissparam.ch/, accessed on 5 January 2025). The system was neutralized with the addition of ions and then equilibrated over 10,000 steps before undergoing energy minimization in 20,000 steps. Next, a classical NVT simulation in molecular mechanics was performed at 1000 ps, followed by NVT equilibration in QM/MM at 2000 steps, adding the NVT-QM/MM.mdp file, the QMatoms as the ligand, and the specifications for the CP2K as follows: qmmm-cp2k-active = true; qmmm-cp2k-qmgroup = QMatoms; qmmm-cp2k-qmmethod = PBE; qmmmcp2k-qmcharge = 0; and qmmm-cp2k-qmmultiplicity = 1. After equilibration, the QM/MM calculation was performed, treating the QM part with the DFT functional set and the DZVP-MOLOPT basis set, and the MM part with the CHARMM27 force field, to provide the binding free energy at 2000 steps using the above specifications in the QM/MM calculation.mdp file and including the mandatory “qmmm-cp2k-qmmethod = INPUT”, adding the option-qmi qm-mm.inp in the command line.

## 3. Results

### 3.1. Synthesis of the Compounds

To obtain novel antileishmanial compounds, compounds from the AMTAC and ACMD series were designed and synthesized. Scheme **1** outlines the synthesis sequence and reactions required to obtain the compounds AMTAC-01, AMTAC-02, AMTAC-06, ACMD-01, ACMD-03, and ACMD-06. As well established in previous studies [42,43], 9-acridinaldehyde reacts in triethylamine and absolute ethanol with the substituted 2-cyano-*N*′-benzylidene-acetohydrazide or 2-cyano-*N*′-phenyl-acetamide intermediates through a Knoevenagel condensation to produce the AMTAC or ACMD derivatives, respectively, that undergo spontaneous cyclization between N1 of hydrazine or amide and C-9 carbon of acridine [23]. The yield was greater than 50% for the final molecules, except for chlorinated compounds (AMTAC-06 = 24% and ACMD-03 = 31%), possibly due to the electronic effects produced by the chlorine atom.

The structures of all new spiro-acridine derivatives (ACMD-01, ACMD-03, and ACMD-06) were determined by ^1^H and ^13^C NMR, IR, and high-resolution mass spectrometry. Through ¹H NMR analysis, characteristic signals were identified as singlets of the spiro-acridine proton (NH), with chemical shifts ranging from δ 9.48 to 9.63 ppm. Additionally, in the spectra of ACMD compounds, compared to the spectra of AMTAC compounds, there is the absence of a signal attributed to the proton of the imine group, with only one singlet between 8.54 and 8.62 ppm. Furthermore, the ^1^H NMR spectra of all derivatives demonstrated characteristic singlet (s), doublet (d), and multiplet (m) for aromatic acridine groups [23,24]. In turn, the ^13^C NMR spectra also confirmed the structures by the presence of signals corresponding to spiro-acridine carbon atoms. A shift of 70.60 ppm was observed, indicating the formation of a quaternary carbon after the spontaneous cyclization process [44].

The infrared results also contributed to the structural characterization of spiroacridines. The following bands were observed: 3304.07–3314.63 cm^−1^, suggestive of the axial deformation of the NH; a band between 2235.35 and 2242.08 cm^−1^, suggestive of C≡N; and a band between 1679.24 and 1695.14 cm^−1^, suggestive of the aromatic axial strain of C=O [23,24]. Ultimately, mass spectrometry (MS) was helpful in the structural characterization of the new spiro-acridine derivatives. These data are present in the Supplementary Materials.

### 3.2. Biological Evaluation

#### 3.2.1. Anti-Promastigote Activity

The evaluation of the leishmanicidal potential of spiro-acridine derivatives from the AMTAC and ACMD series was initially conducted by determining their anti-promastigote effects against the *L. amazonensis* and *L. infantum* species, which cause cutaneous and visceral leishmaniasis, respectively. In this assay, the commercial drug Amphotericin B (ANFO B) was used as a positive control (Table 1).

The compounds showed good activity against both parasitic strains, especially the AMTAC molecules against *L. amazonensis* promastigotes (0.73–1.23 µM). Additionally, none of the spiro-acridine compounds showed toxicity in human red blood cells when tested up to a concentration of 400 µM, demonstrating higher safety of the tested compounds in comparison to the control drug used in leishmanicidal therapy, amphotericin B, which presented an HC_50_ of 24.25 µM, confirming reports in the literature about its hematological toxicity, which can lead to anemia and leukopenia in patients treated with this medication [45,46].

Furthermore, it is observed that, in general, the compounds showed better results against promastigote forms of *L. amazonensis*, especially the AMTAC compounds. Therefore, these were evaluated against amastigote forms of *L. amazonensis* to determine their full anti-leishmanial activity. Accordingly, the most active compounds from each series (AMTAC-02 and ACMD-03) were selected.

#### 3.2.2. Anti-Amastigote Evaluation

The evaluation of anti-amastigote activity in *L. amazonensis* and toxicity in J774 macrophages was carried out with the spiro-acridines AMTAC-02 (-OCH_3_) and ACMD-03 (-Cl), which demonstrated the best results in the anti-promastigote assay against the respective species (IC_50PRO_ = 0.73 µM and 10.95 µM, respectively) (Table 2).

Both compounds showed promising anti-amastigote activity (IC_50AMA_ of 13.50 µM and 10.47 µM), with ACMD-03 being more active than AMTAC-02, as demonstrated by its IC_50_ value being slightly lower than that obtained in the anti-promastigote assay.

Regarding the cytotoxicity evaluation in macrophages, both compounds proved to be less toxic than the reference drug, with CC_50_ of 569.50, 27.22, and 3.09 µM for AMTAC-02, ACMD-03, and the reference drug, amphotericin B, respectively. Hence, although the compound ACMD-03 demonstrated better anti-amastigote activity than AMTAC-02, it also demonstrated more significant toxicity in macrophages, which, consequently, resulted in a lower selectivity index (SI = 2.6) when compared to AMTAC-02 (SI = 39.6) and ANFO B (SI = 2.8).

#### 3.2.3. Cytokine Profile of Immunological Responses

To determine possible mechanisms of action that could justify the leishmanicidal effect developed by spiro-acridine compounds, they were evaluated for their ability to modulate Th1, Th2, and Th17 responses.

**Figure 1:**
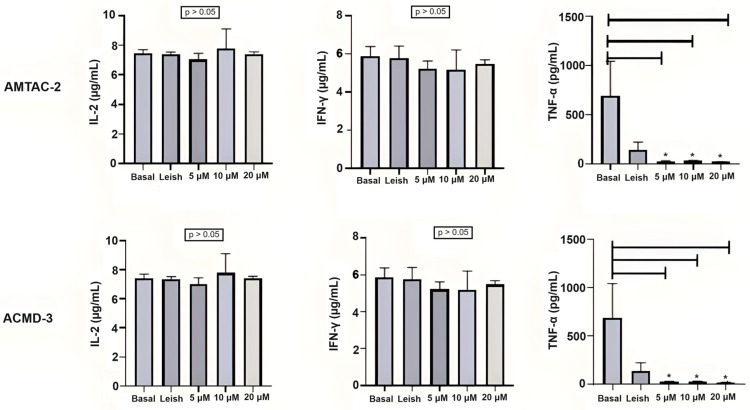
Assessment of the Th1 immune response through IL-2, INF-γ, and TNF-α levels. Basal: Macrophages uninfected and untreated. Leish: Macrophages infected with *L. amazonensis* and untreated. Asterisks represent statistical differences between treated and untreated infected macrophages; * *p* < 0.05. ANOVA followed by Tukey’s multiple comparisons test.

The spiro-acridines AMTAC-02 and ACMD-03 did not demonstrate a modulation of the IL-2 response in basal macrophages or macrophages infected with *L. amazonensis* at the concentrations tested (2, 4, and 8 µg/mL). On the other hand, a significant reduction in TNF-α expression, caused by both molecules at the three tested concentrations, was observed in infected macrophages, which may indicate a reduction in the Th1 response. The statistical significance between infected macrophages treated and untreated with spiro-acridine derivatives is indicated by asterisks (* *p* < 0.05) (Figure 1).

**Figure 2:**
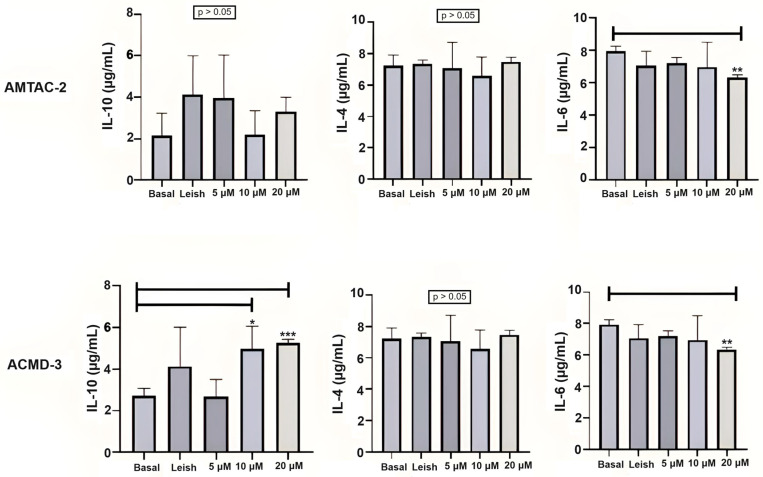
Assessment of the Th2 immune response through IL-10, IL-4, and IL-6 levels. Basal: Macrophages uninfected and untreated. Leish: Macrophages infected with *L. amazonensis* and untreated. Asterisks represent statistical differences between treated and untreated infected macrophages; * *p* < 0.05, ** *p* < 0.01, *** *p* < 0.001. ANOVA followed by Tukey’s multiple comparisons test.

For the Th2 response, the compound ACMD-03 significantly increased the expression of IL-10 at concentrations of 4 and 8 µg/mL. On the other hand, there was no significant increase in IL-10 expression by treatment with AMTAC-02. Neither of the two molecules significantly influenced IL-4 expression at the concentrations tested. Nonetheless, a significant decrease in IL-6 expression was observed in macrophages treated with both molecules at a concentration of 8 µg/mL in comparison with macrophages infected with *L. amazonensis* and untreated (** *p* < 0.01) (Figure 2).

**Figure 3:**
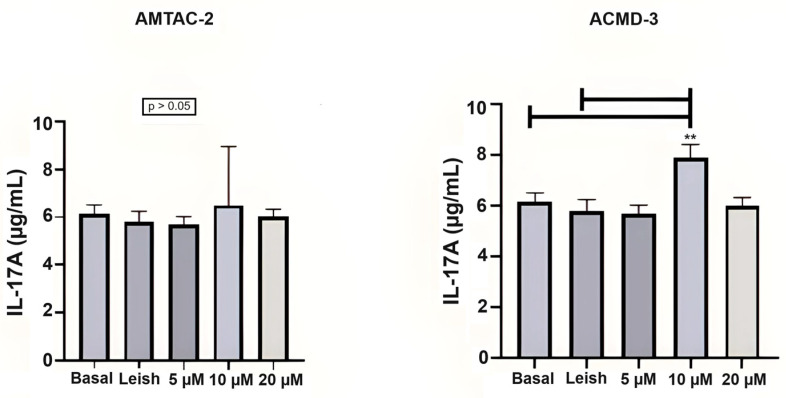
Assessment of the Th17 immune response through the levels of IL-17A. Basal: Macrophages uninfected and untreated. Leish: Macrophages infected with *L. amazonensis* and untreated. Asterisks represent statistical differences between treated and untreated infected macrophages; ** *p* < 0.01. ANOVA followed by Tukey’s multiple comparisons test.

The evaluation in Th17 cells showed that ACMD-03 demonstrated a significant increase in this interleukin at a concentration of 4 µg/mL concerning basal and parasite-infected macrophages (** *p* < 0.01), demonstrating a possible modulatory effect on the Th17 response (Figure 3).

Although AMTAC-02 and ACMD-03 did not demonstrate efficient immunomodulation in T helper responses to control leishmaniasis caused by *L. amazonensis* in the tests carried out, it is essential to highlight that these spiro-acridine derivatives showed important leishmanicidal activity in both promastigote and amastigote forms of the parasite, which can suggest leishmanicidal action through mechanisms of action other than immune regulation through T helper cells.

#### 3.2.4. Reactive Oxygen Species (ROS) and Reactive Nitrogen Species (RNS) Assays

**Figure 4:**
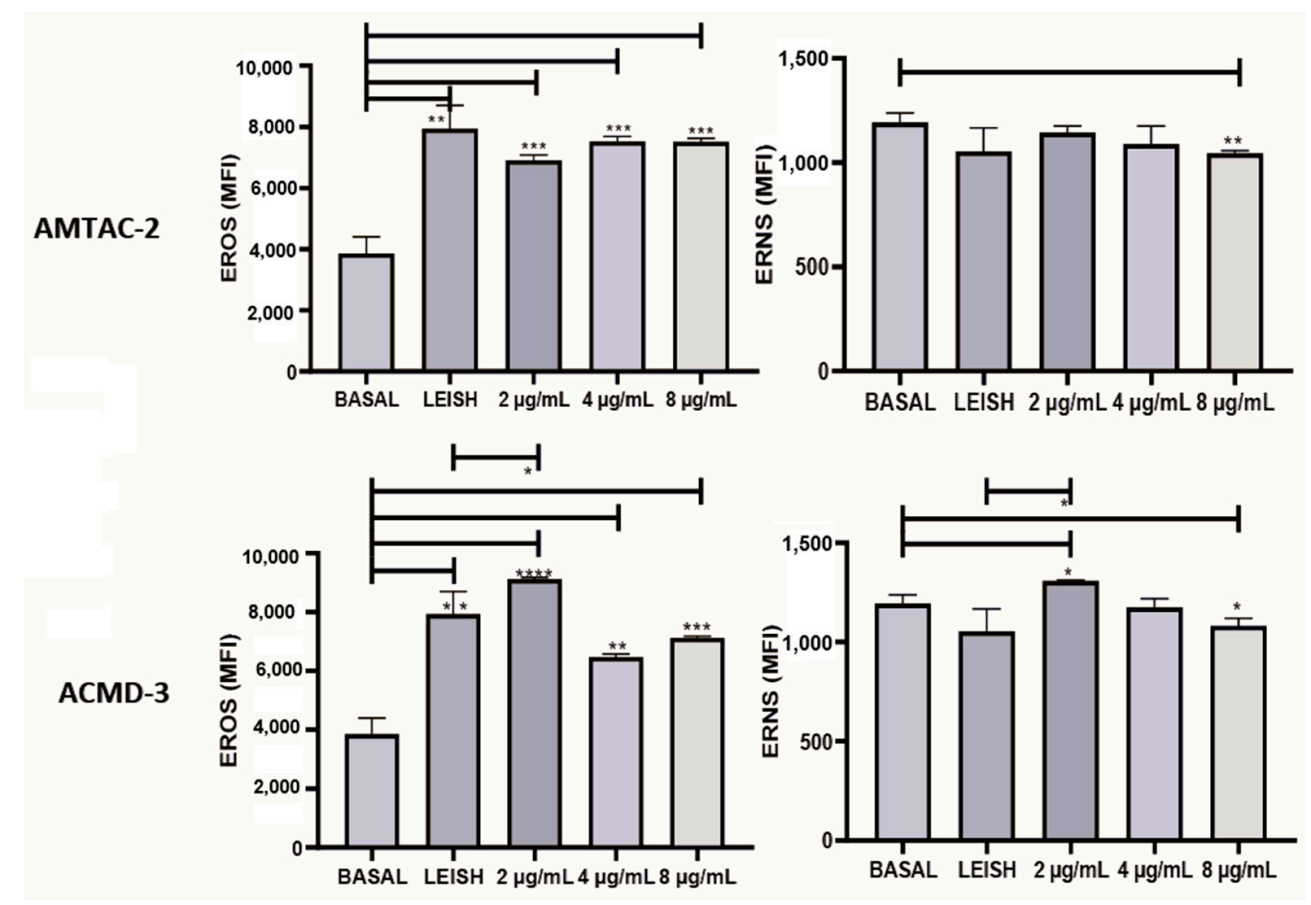
Evaluation of the expression of reactive oxygen species (ROS) and nitrogen species (RNS) of the spiro-acridine derivatives AMTAC-02 and ACMD-03. Leish: Macrophages infected with *L. amazonensis* and untreated. Asterisks represent statistical differences between treated and untreated infected macrophages; * *p* < 0.05, ** *p* < 0.01, *** *p* < 0.001, and **** *p* < 0.0001. ANOVA followed by Tukey’s multiple comparisons test.

The production of reactive oxygen species (ROS) and reactive nitrogen species (RNS) is of substantial relevance to disease progression. Thus, to evaluate the ability of the synthesized compounds to induce the formation of reactive species, they were tested at concentrations of 2, 4, and 8 µg/mL. As a result, it was observed that both significantly increased (*p* < 0.01) the expression of ROS at all concentrations tested compared to the basal macrophage. In addition, spiro-acridine ACMD-03 significantly increased (* *p* < 0.05) RNS at a concentration of 2 µg/mL concerning the infected macrophage, demonstrating that this molecule significantly increases the expression of both types of reactive species (Figure 4).

### 3.3. Molecular Modeling Studies to Propose the Biological Target

#### 3.3.1. Molecular Docking

Initially, protocol validation was necessary to perform molecular docking and select the optimal scoring function. Thus, the re-docking was performed, and RMSD values were calculated (Table S1, see Supplementary Materials). For TyR (trypanothione reductase from *Leishmania infantum*), the best score function was ChemPLP (RMSD of 3.02), and for CYP51 (sterol 14α-demethylase from *Leishmania infantum*), GoldScore was chosen (RMSD of 1.86). Topo I (topoisomerase I of *Leishmania donovani*) and CPBLa (cysteine protease B from *Leishmania amazonensis*) do not show cocrystallized ligands, and ChemPLP was selected because it presented the best-fit score value.

**Figure 5:**
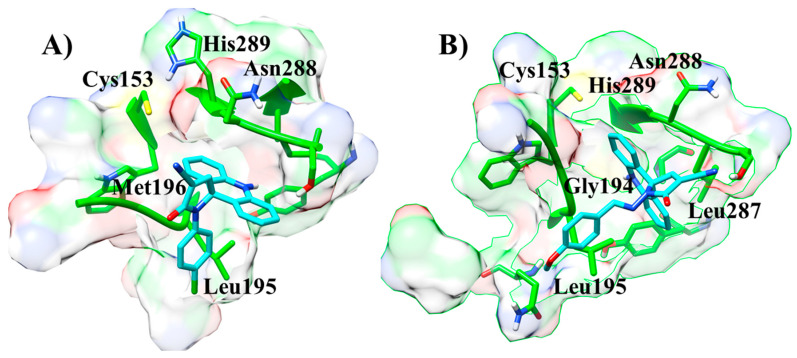
Interactions at the active site of CPBLa of the compounds ACMD-03 (**A**) and AMTAC-02 (**B**).

Next, the molecular docking was performed using AMTAC-02 and ACMD-03 to propose the biological target (Table 3). In fact, the best-fit score was against the CPBLa, and, similar to biological assays, the compound ACMD-03 (50.05) provided the best results compared to AMTAC-02 (44.71) and the standard compound (1c, 2-hydroxy-4-O-(3,3-dimethylallyl) benzophenone) (47.77). In addition, both compounds showed significant interactions at the active site of CPBLa (Figure 5). The main interactions for both were π-alkyl with Leu^287^ and Leu^195^, amide-π with Asn^288^, and the interactions with the catalytic residues His^289^ (van der Waals) and Cys^153^ (π-sulfur). Additionally, AMTAC-02 forms an H-bond with Gly^194^ (Figure 5A), and ACMD-03 forms a π-sulfur interaction with Met^196^ (Figure 5B). These interactions are similar to those with the standard compound (such as with the catalytic residues) and can suggest that CPBLa is the primary drug target related to its antileishmanial activity.

#### 3.3.2. Molecular Dynamics Simulations and MM-PBSA Calculations

**Figure 6:**
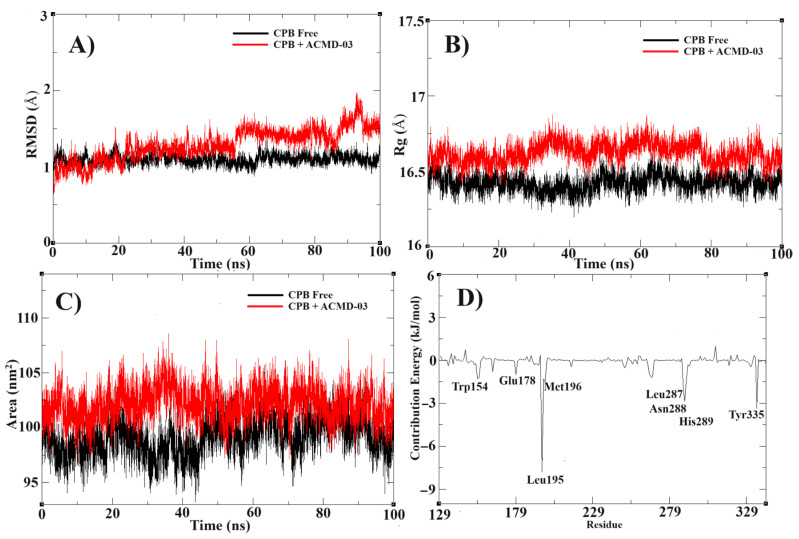
Molecular dynamics simulations results, showing the RMSD (**A**) and R_g_ (**B**), SASA (**C**), and contribution energy per residue (**D**) of the CPB free (black line) compared to CPB in complex with ACMD-02 (red line).

Following molecular docking, a molecular dynamics simulation study was conducted to confirm the stability of the proposed target complex. In this way, the MD simulation at 100 ns shows high stability of the ACMD-03 at the binding site of CPBLa. First, the RMSD of the free CPBLa and in complex with ACMD-03 (Figure 6A) shows similar stability from 60 ns, remaining until the end of the simulation, with minimal variations, up to 2 Å. In addition, the experiment of radius gyrate (R_g_) (Figure 6B) shows a similar standard of free CPB and in complex with ACMD-03, with variations around 16.5 Å, providing that the ligand does not interfere with the rigidity and compactness of the protein. Next, the SASA plot was generated and provided a similar standard in both, showing that the water molecules do not interfere with the stability of the complex. Similar to other works [26,48], these findings provide the stability of the complex and propose the CPB as the primary drug target of this class of compounds.

**Figure 7:**
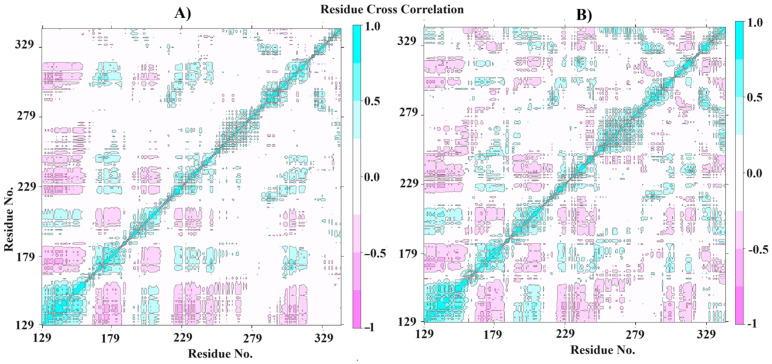
Dynamics cross-correlation matrix (DCCM) of the CPBLa free (**A**) and in complex with ACMD-03 (**B**).

The MM-PBSA calculations were performed to highlight the free binding energy of the interaction and the per-residue contribution. In this way, our findings suggest that the residues Leu^195^, Met^196^, Leu^287^, Asn^288^, His^289^, and Tyr^335^ are the most related to the coupling process (Figure 6D), with the best values of contribution energy (between −1.1542 and −7.8202 kJ/mol). In fact, the residues Leu^195^ and Tyr^335^ make the best energetic contributions (−7.8202 and −3.3774 kJ/mol, respectively) and can be explored further in drug design studies. In addition, the MM-PBSA (Table 4) confirms the high affinity of ACMD-03 to CPBLa, showing a ΔGbind of −65.225 kJ/mol, which is similar to other works [38,49]. Furthermore, van der Waals interactions are the primary interactions related to the maintenance of the complex (−107.611 kJ/mol) compared to the electrostatic interactions (−4.489 kJ/mol), which are expected due to the scaffold of acridine. Finally, the plot of the dynamic cross-correlation matrix (DCCM) (Figure 7) was generated to confirm the abovementioned findings. Thus, comparing the plots of CPBLa free (Figure 7A) and in complex with ACMD-03 (Figure 7B), the correlated movements (cyan) are more highlighted, with the residues between 130–160 and 280–300, in which are included the Cys^153^, Leu^287^, Asn^288^, His^289^, and Tyr^335^ identified in MM-PBSA calculations. Once again, our findings suggest that these residues can be utilized in further drug design work against this target.

**Figure 8:**
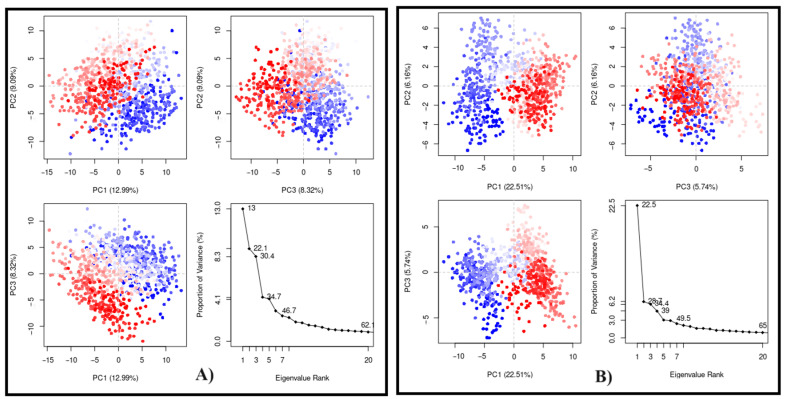
Principal component analysis (PCA) of the MD simulation of CPBLa (**A**) and in complex with ACMD-03 (**B**). The blue and red dots represent the main conformational states, and the white dots represent the intermediate. Each point represents a protein conformation.

Finally, the PCA plots (Figure 8) were obtained after 100 ns of MD simulation in a two-dimensional system using the first three eigenvectors (PC1, PC2, and PC3) to evaluate the conformational states. In this way, the three first components are related to the most significant structural variations. PC1 was the most important for both, showing 12.99% for CPBLa free (Figure 8A) and 22.51% in the complex (Figure 8B). In addition, the uniformity of the points highlighted suggests that the system is stabilized to a similar standard for both. These data corroborate with other experiments, such as RMSD and R_g_, suggesting the stability of the ACMD-03 at the binding site of CPBLa, which proposes the protein as the primary drug target of this class of compounds.

#### 3.3.3. QM-MM Calculations

Finally, to confirm our findings and re-score the ACMD-03, proposing the CPBLa as a drug target, we performed a QM-MM calculation. In this way, the ligand was treated as quantum portion (QM) and the protein as the molecular mechanics (MM). Here, using GROMACS with a CP2K interface, we calculated the QM-MM at 2000 steps for the ACMD-03 in complex with CPBLa and compared it with the standard compound. In this way, ACMD-03 exhibits the best QM-MM energy (−199.30 hartree) compared to the standard compound (−176.72 hartree), confirming it as a CPBLa inhibitor. Our findings are similar to those of other works that utilized QM-MM to provide the energy interaction and suggest the binding of the ligand to the target [50,51]. Once again, ACMD-03 shows the best result compared to the standard compound, and these findings propose that CPBLa can be the primary drug target of this compound.

## 4. Discussion

This research was based on the potential demonstrated by spiro-acridine derivatives evaluated by Almeida et al. [22] against *L. infantum*, with the aim of obtaining new acridine compounds as candidates for leishmanicidal drugs. The structure of the compound **AMTAC-01** served as a prototype for the design of a simpler spiro-acridine, characterized by the absence of an imine functional bond, which led to the development of ACMD-01. Similar to the design strategies used in obtaining the AMTAC compounds, previously synthesized by Almeida et al. [23] and Gouveia et al. [24], the following work used the Topliss decision tree to determine the substituents for the benzene ring to establish a structure–activity relationship considering the influence of substituents and the imine functional group on the development of the leishmanicidal activity.

Both series of molecules have demonstrated promising leishmanicidal activity when evaluated against promastigote strains of *L. amazonensis* and *L. infantum,* pathogens responsible for the distinctive clinical manifestations of cutaneous and visceral leishmaniasis, respectively.

As previously mentioned, the unsubstituted and chlorinated AMTAC compounds (AMTAC-01 and -06, respectively) were previously evaluated by Almeida et al. [22] in promastigote forms of *L. infantum* employing a different methodology and, similar to the results obtained in this assay, satisfactory results were obtained and demonstrated a decrease in the anti-promastigote activity caused by the insertion of the chlorine atom in the *para*- position of the unsubstituted aromatic ring. According to the Topliss decision tree, in this situation, it is recommended to replace the chlorine atom with a methoxy group to enhance the pharmacological activity due to the modifications in the hydrophobic, electronic, and steric effects [52].

Thus, in AMTAC-02, a methoxyl group was introduced at the para position of the phenyl ring, resulting in a significant enhancement of its anti-promastigote activity against both Leishmania species (IC_50PRO_ of 2.22 µM and 0. 73 µM for *L. infantum* and *L. amazonensis*, respectively) when compared with the unsubstituted compound. Consequently, AMTAC-02 emerged as the most promising candidate among the tested AMTAC molecules, indicating that the methoxyl group plays a vital role in this biological activity, potentially due to its electron-donating effect, which can favor interaction with the biological target.

Ortalli et al. [53] also suggested the importance of the methoxyl group when they synthesized a series of 31 chalcone derivatives (1,3-diaryl-2-propen-1-ones) and acknowledged that the insertion of this function at the C-2 position and the presence of nitro substituent affect the inhibitory behavior against Leishmania. This resulted in derivative 6, which exhibited the best IC_50_ value (3 µM) against the promastigote forms of *L. donovani*. Likewise, the authors observed that the insertion of halogens, such as bromine, substantially reduced the anti-promastigote activity of this compound (IC_50PRO_ = 16 µM) in the same species.

As previously noted, the reduction of anti-promastigote activity by the addition of halogen groups was also verified in this work when analyzing the spiro-acridine derivatives against *L. infantum* since the two compounds substituted with chlorine at the para position of the phenyl, ACMD-03 and AMTAC-06, were those with the lowest anti-promastigote activity against the respective species with IC_50PRO_ values of 7.86 µM and 13.8 µM, respectively. Similar results were observed by Oliveira et al. [54] through the analysis of the anti-promastigote activity of five thiazopyridines derivatives (TP) against *L. infantum*, in which the substitution with the methoxy group (TP-06, IC_50_ < 2.78 μM) maintained the anti-promastigote activity in comparison to the unsubstituted compound (TP-01, IC_50_ < 2.78 μM). Contrarily, the compounds TP-04 (IC_50_ = 7.01 ± 3.72 μM), TP-05 (IC_50_ = 22.55 ± 0.97 μM), and TP-09 (IC_50_ = 5.52 ± 0.29 μM), substituted with halogens (Br, F, and Cl, respectively), were less potent than TP-01.

Accordingly, compounds AMTAC-02 and ACMD-03 were selected for the evaluation of their anti-amastigote potential against strains of *L. amazonensis.* The amastigote forms are found within the macrophages of vertebrate hosts, responsible for the most virulent phase of the disease by increasing until provoking the host cell membrane to rupture and being phagocytosed by healthy macrophages, perpetuating the infection in the host [9,55]. The activity of the AMTAC-02 compound against the amastigote form of *L. amazonensis* was lower than what was performed in the anti-promastigote assay, possibly due to the intracellular location of these morphological forms. However, both compounds showed promising anti-amastigote activity, especially ACMD-03.

The AMTAC series differs from the ACMD series by the presence of an imine group (HC=NR) in its structure. The imine functional group possibly increases the distance between the spiro-pyrrole nucleus and the phenyl group. It may act as a Michael donor and/or acceptor, promoting interactions with biological targets and increasing the molecular volume. Therefore, the presence of this group could justify the superior anti-promastigote activity of AMTAC-02 against both strains tested, with values of IC_50PRO_ of 2.22 µM for *L. infantum* and 0.73 µM for *L. amazonensis*. The relevance of the imine function for anti-promastigote activity in *Leishmania* sp. is corroborated by investigations performed by Oliveira et al. [54] with thiazopyridine derivatives and by Silva et al. [56] with thiosemicarbazone compounds, which reinforces the importance of this function in the design of new drugs with leishmanicidal activity.

Therefore, AMTAC-02 deserves to be highlighted for its antileishmanial potential. It has demonstrated high activity in promastigote and amastigote forms, lower cytotoxicity in red blood cells and macrophages, and high selectivity for parasitic cells.

Moreover, to determine the possibility of an immunomodulatory effect, the compounds’ impact on the expression of pro- and anti-inflammatory cytokines was evaluated. The host’s immune response to infection by *Leishmania* sp. involves a series of complex mechanisms, including the action of numerous components of the innate and adaptive immune systems, which are responsible for either eradicating the infection or enabling its progression [9,10]. In this perspective, the influence of the adaptive immune system is crucial in controlling the immune response against Leishmania parasitic infection, primarily through the action of different subtypes of T cells, which are characterized by producing distinct patterns of cytokines [9,57].

It was observed that the effect evidenced in relation to the Th1 response was due to the decrease in the expression of TNF-α, which indicates an opposite effect to the pro-inflammatory effect. While the Th1 response is associated with host resistance to the parasite, the Th2 response through the expression of IL-4, IL-6, and IL-10 is associated with greater susceptibility to infection [58,59,60]. In this sense, ACMD-03 increased IL-10 expression, as this cytokine is responsible for inhibiting the function of M1 macrophages and Th1 lymphocytes, once more emphasizing a possible pro-leishmanial effect of this spiro-acridine in strains of *L. amazonensis,* but which may be relevant in the regulation of tissue remodeling and wound healing in cutaneous leishmaniasis [61,62]. On the other hand, in visceral leishmaniaisis, IL-10 upregulation may be critical for protective immune suppression, predominantly during the initial stages of the infection, highlighting the importance of timing in the therapeutic modulation of this cytokine [63].

Counteracting the effect observed in IL-10 by ACMD-03*,* both compounds produced an inhibitory effect on IL-6 expression, which promotes Th2 differentiation and simultaneously inhibits Th1 differentiation through independent molecular pathways. Its main role is to block pro-inflammatory responses by blocking the expression of the cytokines responsible for this effect and suppressing leukocyte recruitment to the site of infection [64,65]. Thus, as opposed to previous results found for Th1 cytokines, the decrease in the expression of an anti-inflammatory interleukin will favor a pro-inflammatory effect and, consequently, a leishmanicidal action [26].

Recently, the importance of the response mediated by Th17 lymphocytes in leishmaniasis has been investigated due to its function in eliminating microorganisms through the expression of IL-17A, which is fundamental for the activation and recruitment of neutrophils mediating inflammatory responses, and had its expression increased in the presence of ACMD-03 [57,66,67].

Therefore, although the compounds have induced alterations in the expression of particular cytokines, the leishmanicidal effect generated by interference with these components of the immune response is complex to determine, given the opposite effects generated in Th1 and Th2 responses, as seen in the work of Albino et al. [26], in which the results were not conclusive since the compound under analysis reduced TNF-α, IL-10, and IL-6, leading to antagonist effects. Similarly, AMTAC-02 caused the downregulation of TNF-α and IL-6, while ACMD-03 produced the same response, in addition to the upregulation of IL-10 and IL-17A, creating opposite impacts. Therefore, the leishmanicidal effects observed for both compounds might not depend solely on Th response.

The activation of macrophages in response to an infectious process also induces the overproduction of ROS, including superoxide, hydrogen peroxide hydroxyl radicals, and RNS, such as nitric oxide (NO). AMTAC-02 and ACMD-03 have significantly increased ROS expression, while ACMD-03 also intensified RNS expression. ROS and RNS exhibit high microbicidal capacity, as these species can generate oxidative damage to the parasite’s biomolecules, such as lipids, proteins, and DNA, leading to the loss of membrane integrity, defective replication, and, consequently, cell death [59,68,69,70,71,72].

Similar results were reported by Bortoleti et al. [73] when evaluating the leishmanicidal activity (*L. amazonensis*) and immunomodulatory effect of solidagenone (SOL), one of the main constituents of *Solidago chilensis*. The authors noticed that the treatment with SOL (10–160 μM) inhibited the proliferation of promastigotes (IC_50PRO_ = 34.5 μM) through mechanisms that included a reduction in the production of TNF-α and an increase in the level of ROS. Another relevant study was carried out by Rodrigues et al. [74], in which they described cordiaquinone E (CORe), isolated from the roots of *Cordia polycephala* (Lam.), as an effective inhibitor of promastigotes (IC_50PRO_ = 4.5 ± 0.3 µM) and axenic amastigotes (IC_50AMA_ = 2.89 ± 0.11 µM) of *L. brasiliensis* through an indirect action associated with increased levels of NO and ROS, in addition to direct action through cell death by apoptosis.

Hence, these analyses demonstrated that the two spiro-acridines tested could promote antileishmanial activity by increasing oxidative stress in the parasite. Furthermore, through these in vitro assays, it was possible to note that small structural variations can substantially modify the mechanism of action of these compounds, enabling multi-target action, which makes a complete elucidation of the leishmanicidal mechanisms of the studied spiro-acridines more challenging. Therefore, it is essential to expand studies into other possible mechanisms of action, such as the inhibition of enzymes critical to the parasite’s metabolism (trypanothione reductase and topoisomerase I), mechanisms of membrane destabilization, induction of apoptosis, and virulence factors. Thus, to determine the entirety of the mechanisms of action involved in the leishmanicidal activity due to the assumed multi-target profile of both compounds, we sought to evaluate mechanisms of interaction with potential parasite targets through in silico studies.

From the results obtained by molecular docking and dynamics, we highlight the affinity and stability demonstrated by the compound ACMD-03 in complex with the target cysteine protease of *L. amazonensis*. This protein, in particular, is a proteolytic enzyme that is present in all species at all stages of the parasite’s life cycle and plays an essential role in parasite–host interactions, underscoring its potential as a viable and promising pharmacological target. A higher protein concentration is found in amastigote forms, primarily acting as a virulence factor, given that a decrease in its expression interferes with the success of infection in vertebrate hosts [75,76]. Although the mechanisms by which cysteine protease B influences immune system evasion are not fully elucidated, its ability to induce or suppress the expression of pro- and anti-inflammatory cytokines by modulating the activation of T helper cells is known, especially Th1 [77,78,79]. Thus, it is inferred that the inhibition of this enzyme directly affects the host–parasite response by assisting in the action of the components of the immune system and could be involved in the immunomodulatory effect expressed by ACMD-03, as suggested by our previous in vitro results in T helper cells.

Therefore, considering the results obtained in vitro, in which ACMD-03 more effectively inhibited the growth of *Leishmania* amastigote forms, as well as demonstrated a more significant immunomodulatory effect by influencing the Th1, Th2, and Th17 responses and inducing an increase in the expression of ROS and RNS, we can establish a correlation with the in silico results and suggest CPBLa as the possible pharmacological target responsible for the evidenced leishmanicidal effect.

## 5. Conclusions

The proposed acridine compounds were designed and synthesized, resulting in the development of three new spiro-acridine derivatives based on the structure of the AMTAC-01 compound. These were characterized by spectroscopic and spectrometric techniques, highlighting the absorption peaks present in the ¹³C NMR spectra between δ 68.79 and 70.60 ppm, which are characteristic of quaternary carbons and indicative of the occurrence of spontaneous cyclization to form the spiro ring. When evaluated in vitro, the compounds demonstrated good activity against strains of *L. infantum* and *L. amazonensis* in their promastigote and/or amastigote forms, which allowed the establishment of a structure–activity relationship that emphasizes the presence of the imine bond in conjunction with the p-methoxyphenyl ring as beneficial for leishmanicidal activity (AMTAC-02). In contrast, in the absence of the imine bond, the activity is improved with chlorinated compounds (ACMD-03). The ability of the selected molecules to develop a leishmanicidal effect through the immune system was also observed, mainly due to the increase in the expression of reactive oxygen species at all concentrations tested. Additional molecular docking, dynamics, and QM-MM studies contributed to mechanistic elucidation by evaluating the compounds against targets essential for the survival of the parasite, in which we emphasize the superior affinity of the spiro-acridine derivatives against the topoisomerase IB and CPBLa targets in comparison to the reference compounds, indicating these as possible pharmacological targets. Therefore, the results demonstrate the potential of spiro-acridine compounds and encourage further studies to determine the entirety of the leishmanicidal effect and, consequently, to obtain promising drug candidates.

## Figures and Tables

**Table 1:** Data on 50% growth inhibition of *L. infantum* and *L. amazonensis* promastigotes (IC_50PRO_) and concentration capable of promoting 50% hemolysis (HC_50_).

Compounds	*L. infantum* IC_50PRO_ (µM)	*L. amazonensis* IC_50PRO_ (µM)	HC50 (µM)
AMTAC-01	6.27	1.23	>400
AMTAC-02	2.22	0.73	>400
AMTAC-06	13.8	0.75	>400
ACMD-01	3.90	ND *	>400
ACMD-03	7.82	10.95	>400
ACMD-06	3.50	234.95	>400
ANFO B	0.05	1.53	24.25

* Not determined.

**Table 2:** Data on 50% growth inhibition of *L. amazonensis* axenic amastigotes (IC_50AMA_), 50% cytotoxic concentration in J774 macrophage (CC_50_), and selectivity index (SI).

Compound	IC_50AMA_ (µM)	CC_50_ (µM)	SI
AMTAC-02	13.50	569.50	39.6
ACMD-03	10.47	27.22	2.6
ANFO B	1.08	3.09	2.8

**Table 3:** Molecular docking against potential antileishmanial targets.

Target	Fit Score AMTAC-02	Fit Score ACMD-03	Standard
4APN (TyR)	68.58	60.81	94.51
3L4D (CYP51)	23.84	41.40	63.18
2B9S (Topo I)	52.55	53.00	63.98 *
1TL8 (Topo I human)	64.35	57.91	82.56
CPBLa	44.71	50.05	47.77 *

* Value obtained from previous work [22], and the standard compound **1c** (2-hydroxy-4-*O*-(3,3-dimethyl)-allylbenzophenone) was obtained from [47].

**Table 4:** MM-PBSA to provide free binding energy and interaction parameters.

Interaction Parameter	Value for ACMD-03 (in kJ/mol)
ΔGbind	−65.225 ± 8.181
SASA energy	−11.926 ± 1.205
Polar solvation energy	58.801 ± 8.727
Electrostatic energy	−4.489 ± 6.560
van der Waal energy	−107.611 ± 12.071

## Data Availability

Data are contained within the article and Supplementary Materials.

## Supplementary Materials

The following supporting information can be downloaded at https://www.mdpi.com/article/10.3390/microorganisms13061297/s1, Figure S1: Infrared spectrum of ACMD-01; Figure S2: Infrared spectrum of ACMD-03; Figure S3: Infrared spectrum of ACMD-06; Figure S4: Mass spectrometry of ACMD-01; Figure S5: Mass spectrometry of ACMD-03; Figure S6: Mass spectrometry of ACMD-06; Figure S7: ^1^H NMR spectrum of ACMD-01 (300 MHz, DMSO-d6); Figure S8: ^1^H NMR spectrum of ACMD-03 (300 MHz, DMSO-d6); Figure S9: ¹H NMR spectrum of ACMD-06 (300 MHz, DMSO-d6); Figure S10: ^13^C NMR spectrum of ACMD-01 (75 MHz, DMSO-d6); Figure S11: ^13^C NMR spectrum of ACMD-03 (75 MHz, DMSO-d6); Figure S12: ^13^C NMR spectrum of ACMD-06 (75 MHz, DMSO-d6). Table S1: RMSD calculations for docking validation protocol.

## Abbreviations

The following abbreviations are used in this manuscript:

1H-NMR	Hydrogen Nuclear Magnetic Resonance
13C-NMR	Carbon Nuclear Magnetic Resonance
ASP	Astex Statistical Potential
B.O.D.	Biochemical Oxygen Demand
CBA	Cytometry Bead Assay
ChemPLP	Chemical Piecewise Linear Potential
DCCM	Dynamic Cross-Correlation Matrix
DCF	2′,7′-dichlorodihydrofluorescein
DAF-FM	4-amino-5-methylamino-2′,7′-difluorofluorescein
DMEM	Dulbecco’s Modified Eagle Medium
DMSO-d6	Deuterated Dimethyl Sulfoxide
FBS	Fetal Bovine Serum
IR	Infrared
MALDI-TOF	Matrix-assisted Laser Desorption/-Time-of-flight
MS	Mass Spectrometry
MM-PBSA	Molecular Mechanics Poisson–Boltzmann Surface Area
M.W.	Molecular Weight
MTT	[3-(4,5-dimethylthiazol-2-yl)-2,5-diphenyltetrazolium bromide]
NNN	Novy–MacNeal–Nicolle
NTD	Neglected Tropical Disease
PBS	Phosphate-buffered Saline
PCA	Principal Component Analysis
QM-MM	Quantum Mechanics-Molecular Mechanics
RMSD	Root-mean-square Deviation
Rg	Radius of gyration
SASA	Solvent-accessible Surface Area
TLC	Thin Layer Chromatography
