# Supplementary material for: Synthesis, Biological Evaluation, Molecular Dynamics, and QM-MM Calculation of Spiro-Acridine Derivatives Against Leishmaniasis
Source: Microorganisms. 2025 Jun 2;13(6):1297. doi: 10.3390/microorganisms13061297 (PMC12195250; doi:10.3390/microorganisms13061297)
Supplement: Supplementary file 1 [file microorganisms-13-01297-s001.zip › microorganisms-3648532-supplementary.pdf]

Synthesis, Biological Evaluation, Molecular Dynamics, and QM-MM Calculation of Spiro-Acridine Derivatives Against Leishmaniasis

Sonaly Albino [1,2,†], Michelangela Nobre [2,†], Jamire da Silva [2], Malu dos Reis [2], Maria Nascimento[2], Mayara de Oliveira [3], Tatiana Borges [4], Lucas Albuquerque [4], Selma Kuckelhaus [3], Luis Alves [5], Fábio dos Santos [5], Maria de Lima [6], Igor Nascimento [2,7,*], Teresinha da Silva [1,6] and Ricardo de Moura [2,7,*]

1 Postgraduate Program in Therapeutic Innovation, Federal University of Pernambuco, Recife 50740-570, Brazil; sonaly.albino@hotmail.com (S.A.); teresinha100@gmail.com (T.d.S.)

2 Drug Development and Synthesis Laboratory, Department of Pharmacy, State University of Paraíba, Campina Grande 58429-500, Brazil; maria.veronica@aluno.uepb.edu.br (M.N.); michelysuelleny@gmail.com (M.N.); jamiremuriel@hotmail.com (J.d.S.); malureis_farmacia@hotmail.com (M.d.R.);

3 Morphology Area, Faculty of Medicine-UnB, University of Brasília, Darcy Ribeiro Campus, Brasília 70910-900, Brazil; mayaragco@gmail.com (M.d.O.); selmask@gmail.com (S.K.)

4 Cellular Immunology Laboratory, Pathology Area, Faculty of Medicine, University of Brasília, Darcy Ribeiro Campus, Brasília 70910-900, Brazil; tatianakarlab@gmail.com (T.B.); lucasffriaca@gmail.com (L.A.)

5 Department of Parasitology, Aggeu Magalhães Institute (FIOCRUZ/PE), Recife 50740-465, Brazil; lcalves@cpqam.fiocruz.br (L.A.); brayner@cpqam.fiocruz.br (F.d.S.)

6 Department of Antibiotics, Federal University of Pernambuco, Biosciences Center, Recife 50740-570, PE, Brazil; nenalima.mariadocarmo@gmail.com

7 Postgraduate Program of Pharmaceutical Sciences, Pharmacy Department, State University of Paraíba, Campina Grande 58429-500, Brazil

* Correspondence: igor.n@visitante.uepb.edu.br or igorjsn@hotmail.com (I.N.); ricardo.olimpiodemoura@servidor.uepb.edu.br (R.d.M.); Tel.: +55-8299933-5457 (I.N.)

† These authors contributed equally to this work.

SUPPLEMENTARY MATERIAL

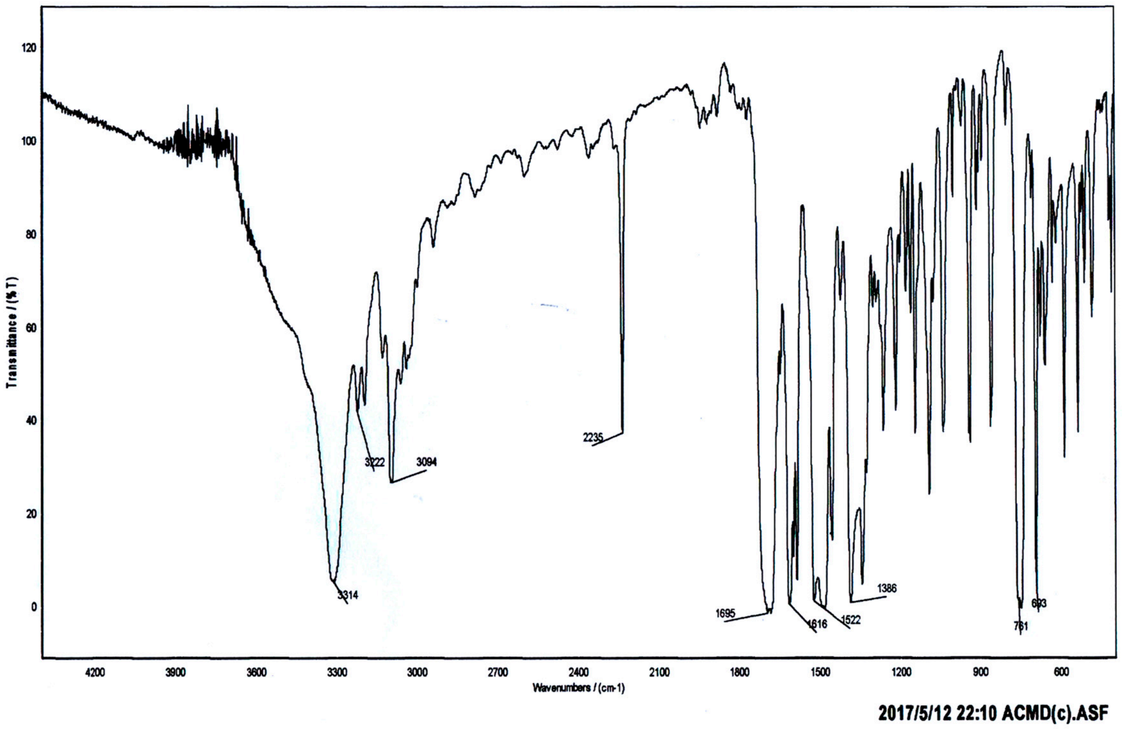

Figure S1. Infrared spectrum of **ACMD-01**

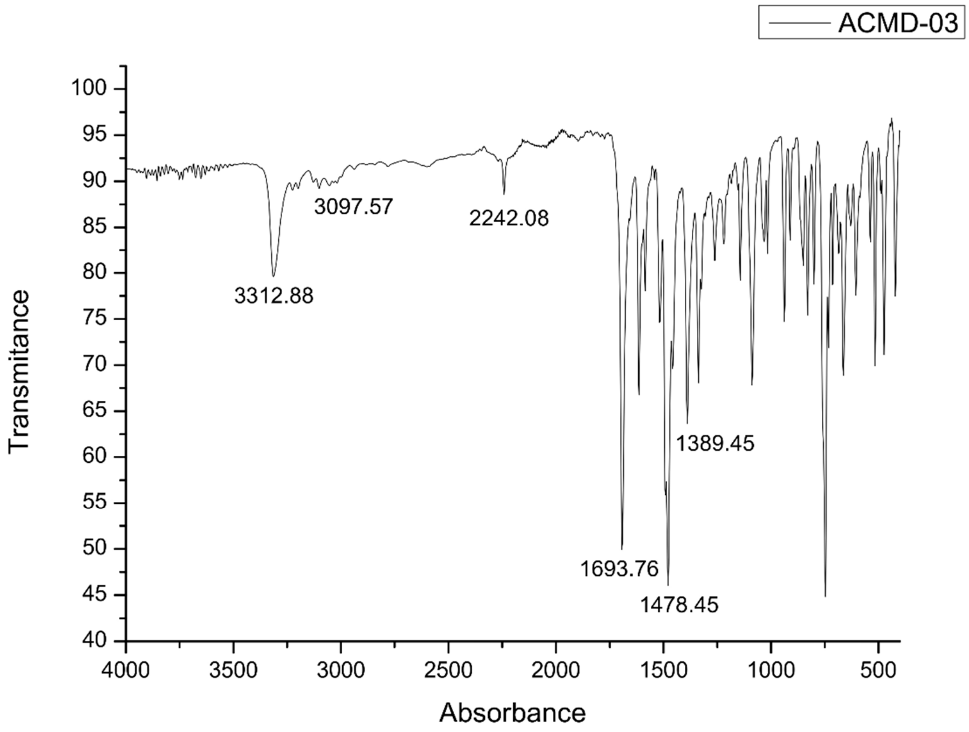

Figure S2 – Infrared spectrum of ACMD-03

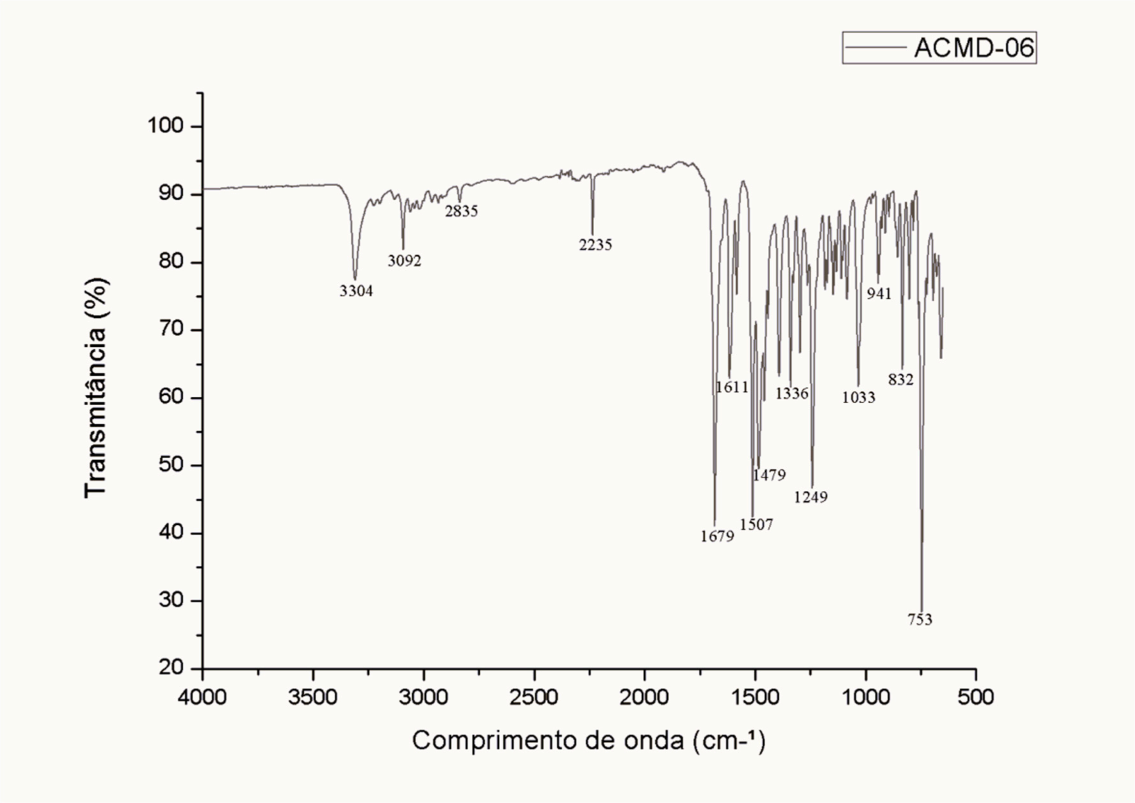

Figure S3 – Infrared spectrum of ACMD-06

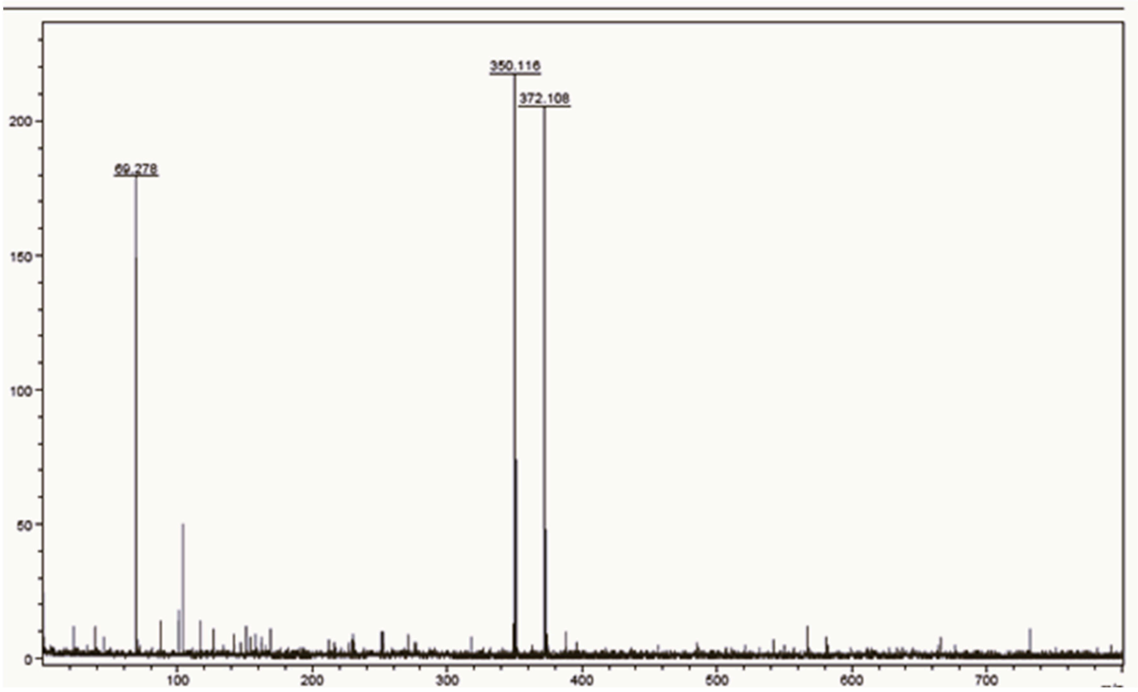

Figure S4 – Mass spectrometry of ACMD-01

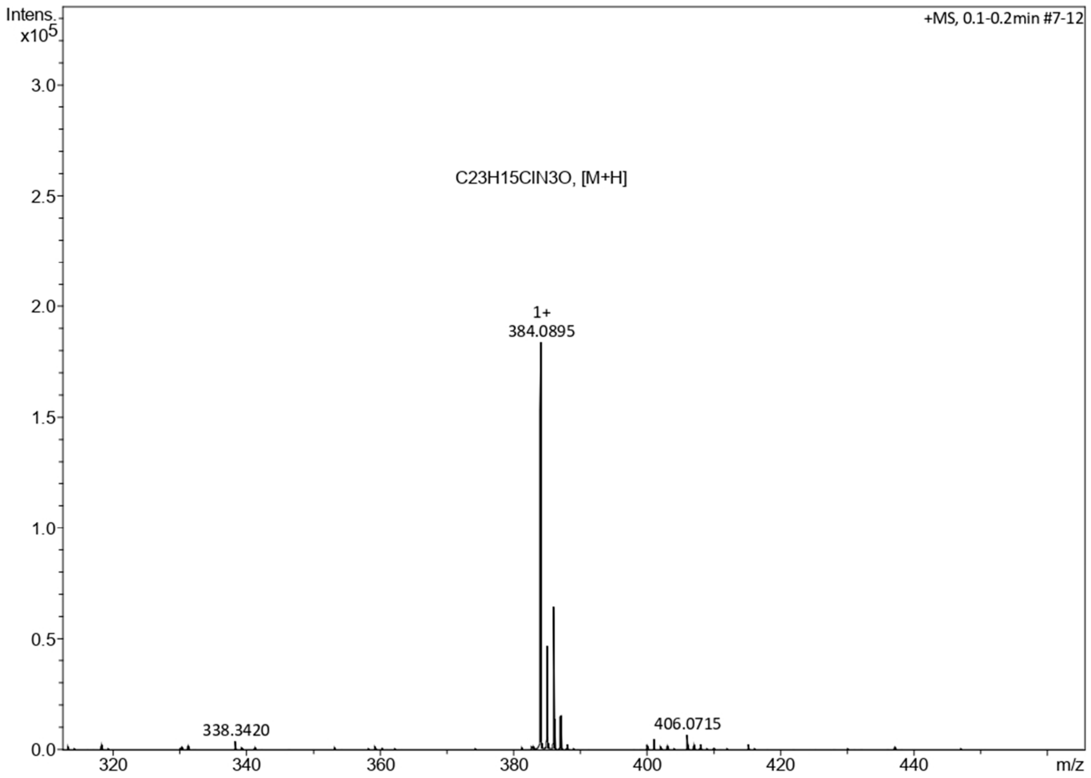

Figure S5 – Mass spectrometry of ACMD-03

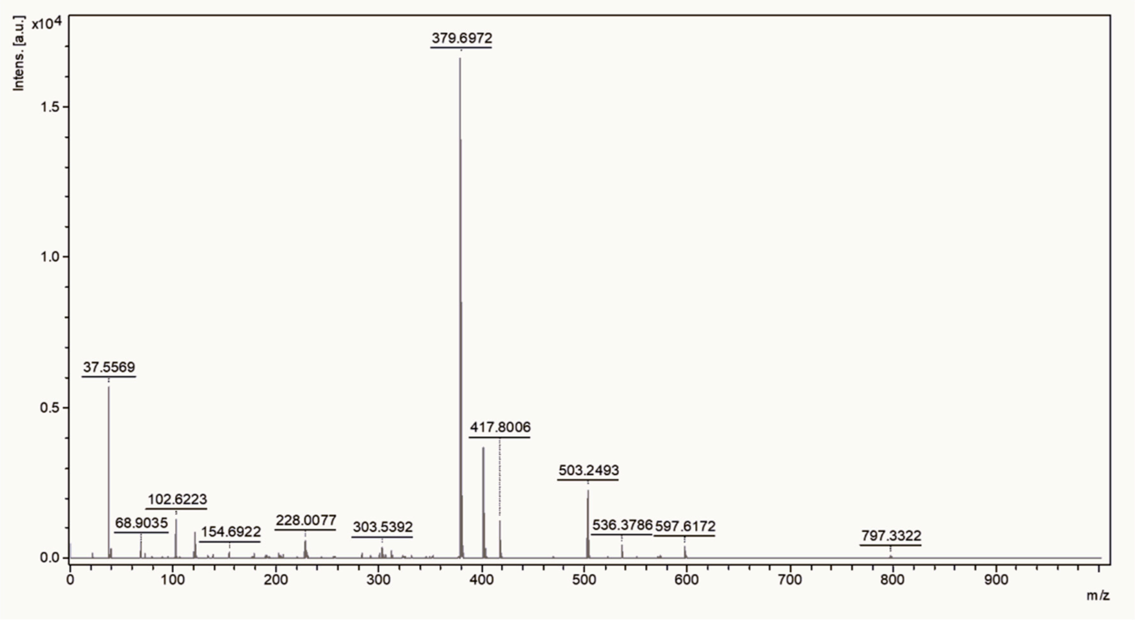

Figure S6 – Mass spectrometry of ACMD-06

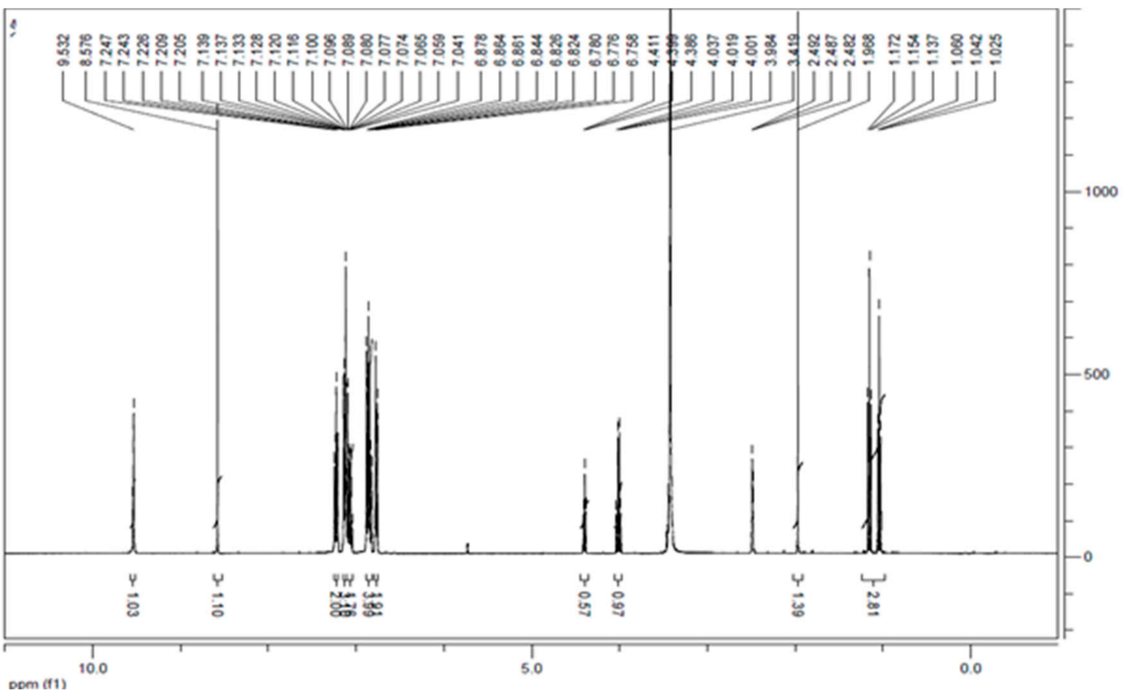

Figure S7 – ^{1}H NMR spectrum of ACMD-01 (300 MHz, DMSO-*d*6)

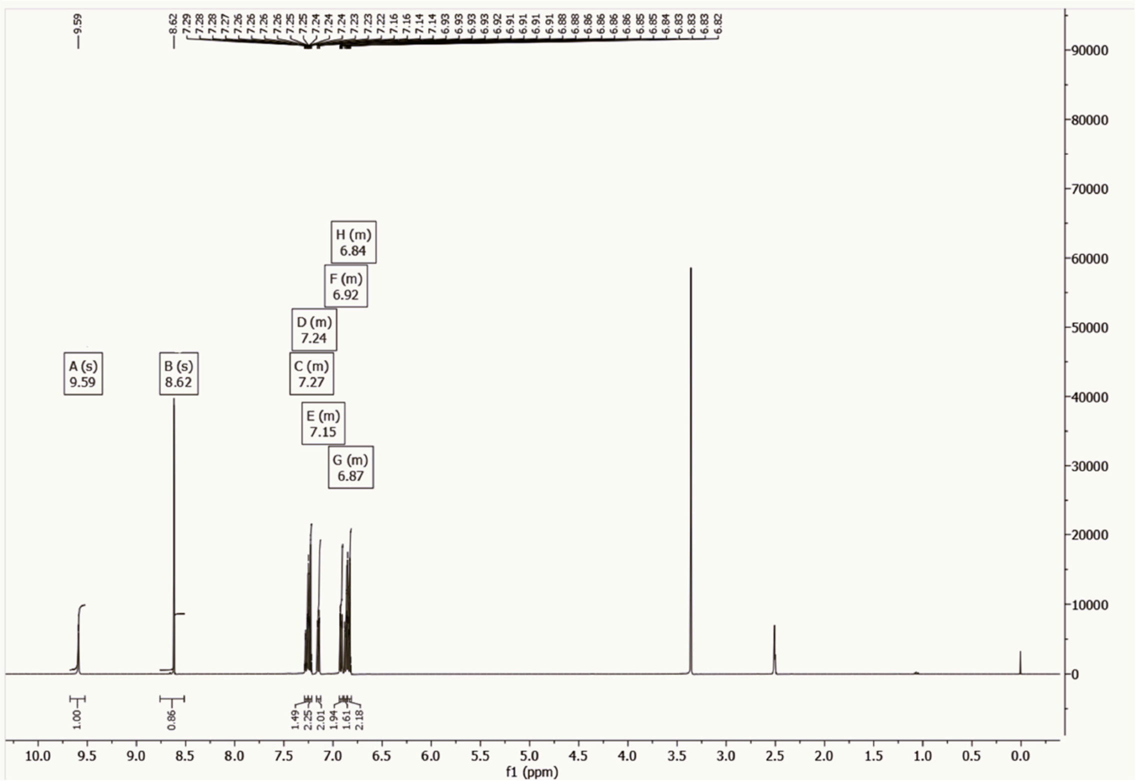

Figure S8 – ^{1}H NMR spectrum of ACMD-03 (300 MHz, DMSO-*d*6)

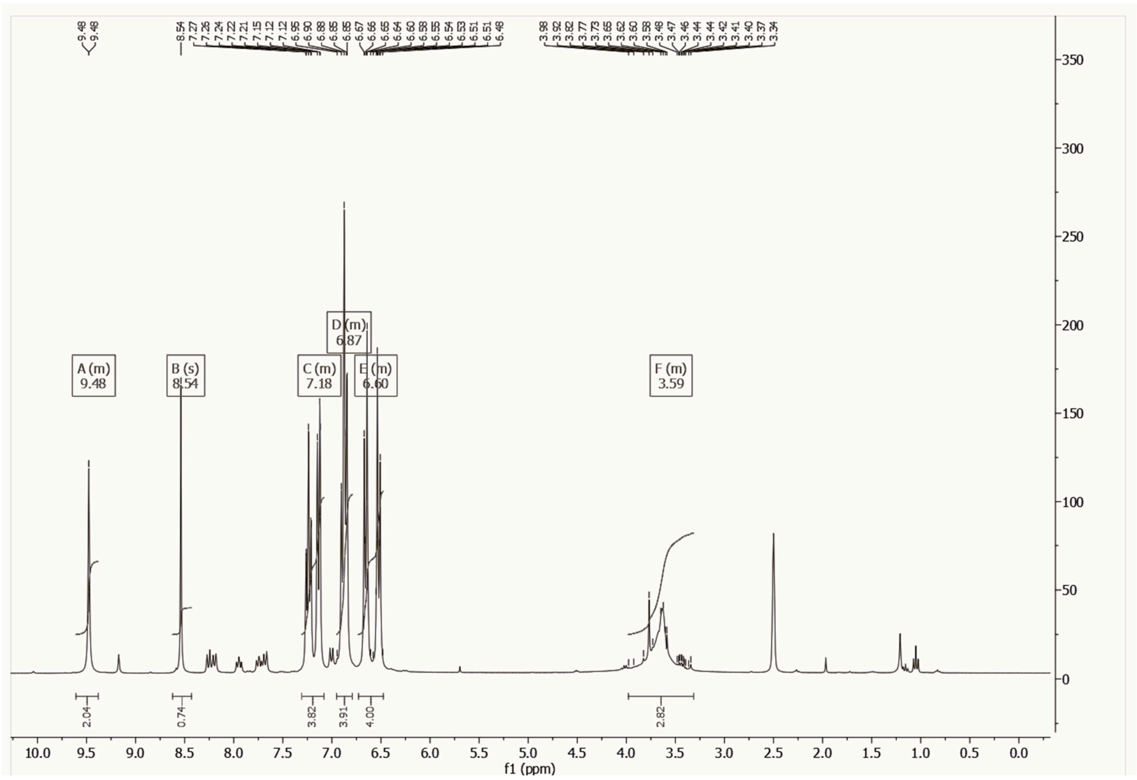

Figure S9 – ^{1}H NMR spectrum of ACMD-06 (300 MHz, DMSO-*d*6)

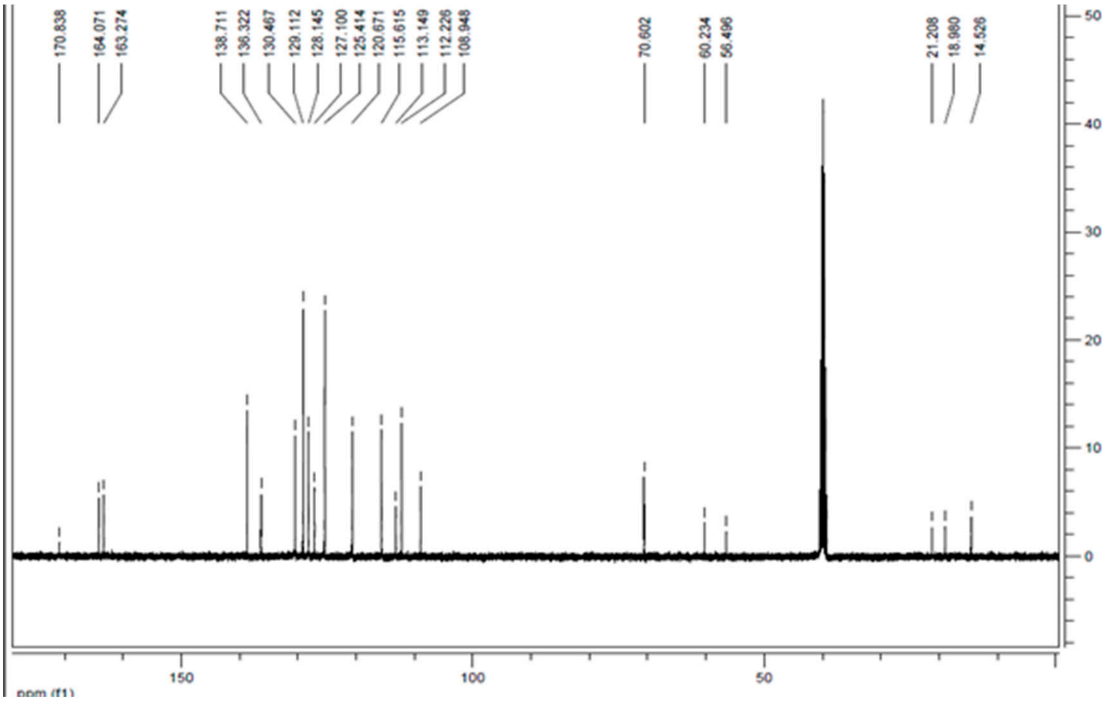

Figure S10 – ^{13}C NMR spectrum of ACMD-01 (75 MHz, DMSO-*d*6)

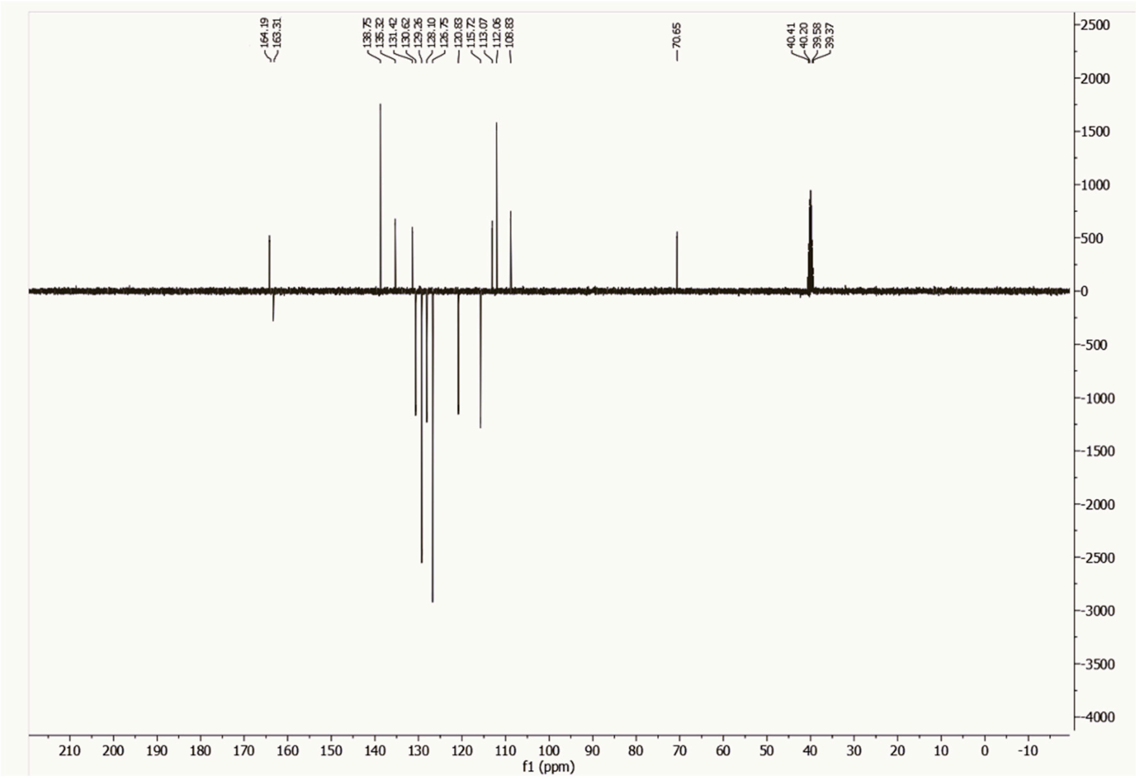

Figure S11 – ^{13}C NMR spectrum of ACMD-03 (75 MHz, DMSO-*d*6)

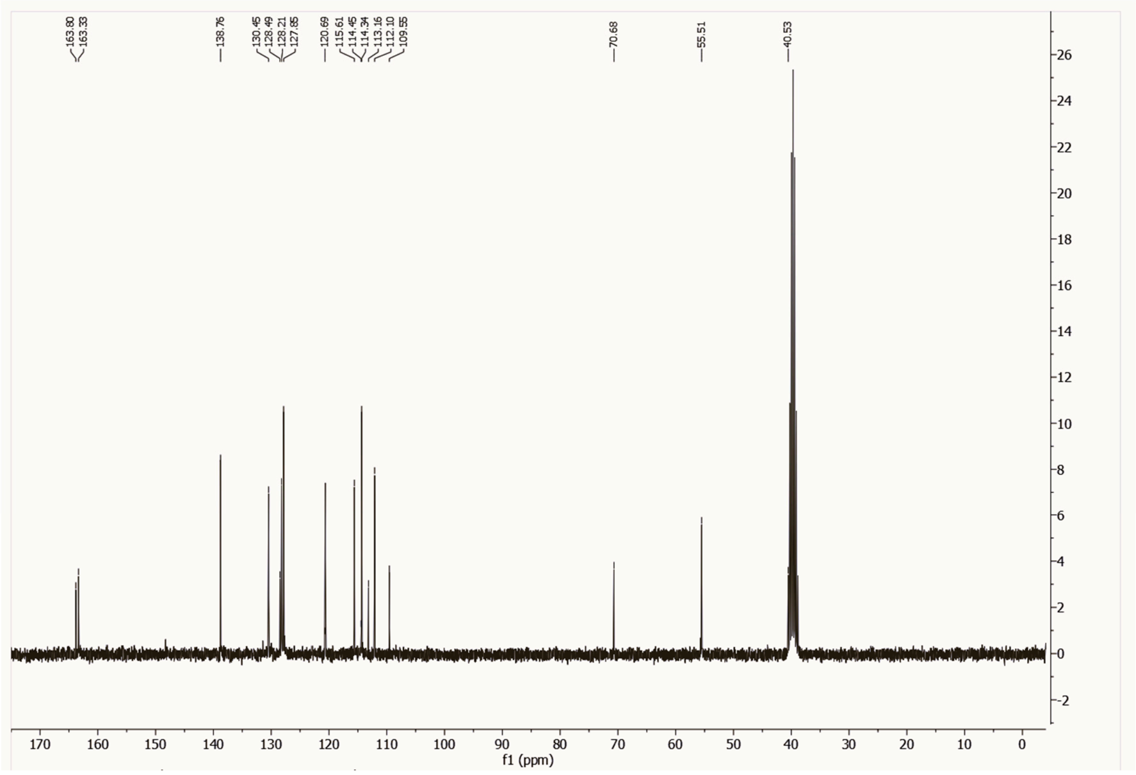

Figure S12 – ^{13}C NMR spectrum of ACMD-06 (75 MHz, DMSO-*d*6)

Table **S1**. RMSD calculations for docking validation protocol.

**PDB ID**	**ChemPLP**		**GoldScore**		**ChemScore**		**ASP**	
	RMSD	Fit score	RMSD	Fit score	RMSD	Fit score	RMSD	Fit score
4APN	3.02	94.51	7.93	67.50	7.38	41.86	3.21	49.43
3L4D	5.43	70.40	1.86	63.18	1.79	17.10	5.43	56.08
2B9S*	-	-	-	-	-	-	-	-
1TL8	0.39	82.56	7.92	89.96	11.74	21.31	1.18	55.05

*Protein structure without ligand cocristalized
